# Emerging Role of l-Dopa Decarboxylase in Flaviviridae Virus Infections

**DOI:** 10.3390/cells8080837

**Published:** 2019-08-05

**Authors:** Efseveia Frakolaki, Katerina I. Kalliampakou, Panagiota Kaimou, Maria Moraiti, Nikolaos Kolaitis, Haralabia Boleti, John Koskinas, Dido Vassilacopoulou, Niki Vassilaki

**Affiliations:** 1 Laboratory of Molecular Virology, Hellenic Pasteur Institute (HPI), 11521 Athens, Greece; 2 Light Microscopy Unit, Hellenic Pasteur Institute, 11521 Athens, Greece; 3 2nd Department of Internal Medicine, Medical School of Athens, Hippokration Hospital, 11521 Athens, Greece; 4 Section of Biochemistry and Molecular Biology, Faculty of Biology, National and Kapodistrian University of Athens, 15701 Athens, Greece

**Keywords:** hepatitis C virus, dengue virus, l-dopa decarboxylase, phosphoinosite-3-kinase (PI3K)

## Abstract

l-dopa decarboxylase (DDC) that catalyzes the biosynthesis of bioactive amines, such as dopamine and serotonin, is expressed in the nervous system and peripheral tissues, including the liver, where its physiological role remains unknown. Recently, we reported a physical and functional interaction of DDC with the major signaling regulator phosphoinosite-3-kinase (PI3K). Here, we provide compelling evidence for the involvement of DDC in viral infections. Studying dengue (DENV) and hepatitis C (HCV) virus infection in hepatocytes and HCV replication in liver samples of infected patients, we observed a negative association between DDC and viral replication. Specifically, replication of both viruses reduced the levels of DDC mRNA and the ~120 kDa SDS-resistant DDC immunoreactive functional complex, concomitant with a PI3K-dependent accumulation of the ~50 kDa DDC monomer. Moreover, viral infection inhibited PI3K-DDC association, while DDC did not colocalize with viral replication sites. DDC overexpression suppressed DENV and HCV RNA replication, while DDC enzymatic inhibition enhanced viral replication and infectivity and affected DENV-induced cell death. Consistently, we observed an inverse correlation between DDC mRNA and HCV RNA levels in liver biopsies from chronically infected patients. These data reveal a novel relationship between DDC and *Flaviviridae* replication cycle and the role of PI3K in this process.

## 1. Introduction

l-dopa decarboxylase (DDC) is the enzyme that catalyzes the decarboxylation of l-3,4-dihydroxyphenylalanine (l-dopa) to dopamine, the first product in the catecholamine biosynthetic pathway. In mammalian tissue, the enzyme is also commonly referred to as aromatic l-amino acid decarboxylase (AADC), since it has been found to decarboxylate other aromatic l-amino acids into biogenic amines, such as serotonin, histamine, and trace amines [1,2,3,4,5,6,7]. Apart from its expression in the nervous system, where it has a well-established role in neurotransmission, DDC has been purified from various peripheral organs, including the liver [8,9,10,11,12,13,14,15]. Although the physiological function of DDC in the periphery remains unclear [16,17,18], alterations in its expression have been reported in several malignancies such as small-cell lung and gastric cancer [19]. In human placenta, a naturally occurring inhibitor of DDC was identified as Annexin V [20]. Interestingly, we have recently reported an interaction of DDC with phosphatidylinositol 4,5 biphosphate 3-kinase (PI3K) in human hepatocytes, kidney, and neuronal cells [21]. PI3K enzymes, acting as catalytic subunit-regulatory subunit dimeric complexes to phosphorylate phosphatidylinositol 4,5-bisphosphate (PIP2) and serine/threonine residues on proteins, are major regulators of metabolism, cell growth, survival, cell migration, and apoptosis [22,23]. Given this central role, viruses often modulate PI3K to increase productive viral replication [24].

The tissue specific expression pattern of DDC and the enzyme structure are incompletely known. The single copy gene encoding for the enzyme is located on chromosome 7 (loci p12.1–12.3) [25] and is composed of 15 exons. The human DDC gene undergoes complex processing leading to the formation of multiple mRNA isoforms [26]. Alternative promoter usage, combined with alternative splicing within the 5′-untranslated region (5′ UTR), gives rise to two different mRNA transcripts, designated neuronal and non-neuronal, but the proteins encoded by these mRNAs are identical [27,28,29,30]. The neuronal type transcript has also been detected in non-neuronal human tissues, such as placenta, kidney, and leukocytes [28,31,32,33] and, more recently, in liver cells [21]. Alternative splicing events have also been reported within the coding region of the gene [34,35]. Among them, an alternative splicing producing a variant lacking exon 3 has been reported [34]. The catalytically active form of DDC is thought to be a holoenzyme with a molecular weight of 100–120 kDa. Several reports have suggested a homodimeric, heterodimeric, or heterotrimeric structure formed by monomers of 40–56 kDa subunits [4,36,37], while later studies identified a homodimer of 50 or 54 kDa [10,11,12,18]. It is assumed that the DDC holoenzyme uses pyridoxal phosphate (PLP) as a cofactor, but the precise structures and functions of the various DDC complexes are unknown.

Dopamine expressed in peripheral tissues has been shown to affect cellular metabolism, proliferation, apoptosis and the progression of certain cancers, including hepatocellular carcinoma (HCC) [38,39,40,41]. More recently, histamine was reported to play a role in hepatitis C virus (HCV) entry [42] and serotonin 2A receptor has been identified as an HCV entry factor [43]. Moreover, in the case of dengue virus (DENV), binding and entry were found to be inhibited by antagonists of the dopamine receptors D2 or D4 [44,45]. However, a role of DDC in viral infections has not yet been established.

Members of the *Flaviviridae* virus family, to which HCV and DENV belong, are major causes of morbidity and mortality worldwide. DENV causes widely distributed and endemic diseases with manifestations in visceral organs and in the central nervous system [46,47,48]. Infections with DENV are acute self-limiting and mostly asymptomatic, but around 25% of infections cause symptoms ranging from mild (dengue fever) to the more severe dengue hemorrhagic fever (DHF) and shock syndrome (DSS) [49]. The viral genome, a positive single-strand RNA, encodes for a polyprotein that is processed into structural (C, prM, E) and non-structural (NS) proteins (NS1, NS2A, NS2B, NS3, NS4A, NS4B, NS5). Viral replication occurs in cells of different organs, including hepatocytes [50,51,52,53]. In contrast, the closely related HCV predominantly establishes persistent infection. It is a major cause of chronic liver disease, with ~71 million individuals at risk of developing liver cirrhosis and HCC [54]. The HCV positive sense, single-stranded RNA encodes for the polyprotein, which is processed into structural proteins (core, E1, and E2), p7 required for assembly and release of virus particles and NS proteins (NS2, NS3, NS4A, NS4B, NS5A, and NS5B) [55,56]. DENV and HCV replication, orchestrated by the viral NS proteins, occurs in endoplasmic reticulum (ER) membrane invaginations or protrusions, respectively [57,58].

Both DENV and HCV interact with the PI3K/AKT pathway to facilitate viral replication and virus spread. At the early stage of infection, DENV activates PI3K signaling to block apoptosis and enhance virus replication [59], whereas at the late stage of infection, DENV promotes cell death [60,61] through downregulation of PI3K/AKT [59,62]. Moreover, PI3K/AKT can regulate DENV infection by promoting cell survival, virus entry, and viral RNA translation [63]. In the case of HCV, a direct effect on PI3K/AKT activation has been shown in infected hepatoma cells [64], mediated by PI3K-NS5A interaction, which protects cells from apoptosis [65,66,67]. Furthermore, based on our previous studies, AKT activation is implicated in HCV [68] and DENV [69] genome replication enhancement, occurring under oxygen tensions that simulate the physiological ones in tissue, i.e. liver hypoxia, in cultured hepatocytes.

Based on our recently reported DDC–PI3K interaction and the role of PI3K/AKT in the HCV and DENV life cycles, here we investigated the possible role of DDC in HCV and DENV replication and virus–host interaction. For this, we employed efficient infectious models, based on hepatocytes adapted to atmospheric or hypoxic (3% *v*/*v* O_2_) conditions, and liver samples from HCV-infected patients. Furthermore, we studied the effect of viral infection on DDC-PI3K complex formation and DDC subcellular distribution in relation to the viral replication sites. Finally, we addressed the implication of PI3K in virus–DDC relationship.

## 2. Materials and Methods

### 2.1. Cell Culture

Huh7 [70], Huh7.5 [71], Huh7-Lunet [72], and VeroE6 cells (originally obtained from ATCC#CRL-1586) were cultured in high glucose (25 mM) Dulbecco’s modified minimal essential medium (Thermo Fisher Scientific, Waltham, MA, USA), supplemented with 2 mM l-glutamine, 0.1 mM non-essential amino acids, 100 U/mL penicillin, 100 µg/mL streptomycin, and 10% (*v*/*v*) fetal calf serum (referred to as complete DMEM). To create oxygen tensions lower than the atmospheric one, cells were cultured in a fully humidified incubator supplied with pure nitrogen gas to reduce oxygen as well as with 5% (*v*/*v*) CO_2_ at 37 °C (New Brunswick CO_2_ incubator; Artisan Technology Group, Champaign, IL, USA) [73].

### 2.2. Human Liver Biopsy RNA Samples

Total RNA from 12 human liver fine-needle biopsy specimens, obtained from patients with chronic HCV infection, was previously isolated and analyzed for HCV RNA and cellular mRNA levels [68]. Sample collection from patients was performed following informed consent and was approved by the Ethical and Scientific Committee of the Hippokration Hospital of Athens (1997/2008). The study was conducted in accordance with the Declaration of Helsinki. 

### 2.3. Viruses and Plasmid Constructs

Plasmids carrying the full-length DV-2 genomes (16681 strain) pFK-DVs and pFK-DVR2A (with a *Renilla* luciferase reporter gene) and plasmids pFK-Jc1 and pFK-i389RLuc2ACore-3′-Jc1 (JcR2a), carrying the full-length HCV genome, have been described previously [74,75]. The subgenomic replicon constructs pFK-sgDVR2A, based on the DV-2 16,681 strain, and pFK_i389LucNS3-3′_dg_JFH (with a *Firefly* luciferase gene), based on the HCV JFH1 strain, have been described previously [74,76]. The mammalian plasmid vector pcDNA3.1(+)-DDC was previously used [77] and contains the full-length cDNA of human DDC cloned after total RNA isolation from human placenta, RT-PCR and digestion of the PCR product and the pcDNA3.1(+) vector with XhoI-HindIII, as described in [33].

### 2.4. In Vitro Transcription

DENV constructs were linearized with XbaI and used for in vitro transcription as described previously [74]. HCV constructs were linearized with MluI and used for in vitro transcription as described previously [78].

### 2.5. Transfection Assays

Electroporation with in vitro transcribed full-length DENV RNAs into Vero E6 cells, subgenomic DENV RNA into Huh7 cells, HCV-derived RNAs into Huh7-Lunet cells, and plasmid vector pcDNA3.1(+)-DDC into Huh7.5 cells was performed as described elsewhere [79]. A capped-polyadenylated *Renilla* luciferase expressing RNA, produced as previously described [80], or the pcDNA3.1+ vector (Invitrogen) were used as negative controls. In brief, 4 × 10^6^ cells were detached by trypsin and resuspended in Cytomix [81] containing 2 mM ATP and 5 mM glutathione, mixed with 10 μg of viral RNA or 20 μg of plasmid DNA and electroporated with a Gene Pulser system (Bio-Rad, Hercules, CA, USA). The cells were immediately transferred to 20 mL of complete DMEM and subsequently seeded as required for the assay.

### 2.6. Preparation of Virus Stocks and Infection Assays

DENV virus stocks were generated in Vero E6 cells as described elsewhere [74] and used to inoculate cells for 4 h. HCV virus stocks were generated as described elsewhere [79] and used to infect naive Huh7.5 cells. For infectivity assays, supernatants from the first round of DENV or HCV infection were used to infect naïve cells.

### 2.7. Virus Titration in Cell Culture Supernatants

DENV virus titers were determined by standard plaque assay (PFU) on target VeroE6 cells as previously described [82]. In short, Vero E6 cells were seeded at 2 × 10^5^ cells per well in 24-well plates and incubated overnight. Cells were infected with 10-fold serial dilutions of virus stocks and incubated for 1 h. The inoculum was removed, and the plates were overlaid with 1.5% carboxymethylcellulose (Sigma-Aldrich, Taufkirchen, Germany) in minimal essential medium (MEM) (Thermo Fisher Scientific, Waltham, Massachusetts, USA). Plates were incubated for seven days and then were fixed with 10% formaldehyde and stained with 1% crystal violet (Sigma-Aldrich, Taufkirchen, Germany) in 10% methanol for 20 min to visualize plaques. Viral titers were expressed as plaque forming units (PFU)/mL. HCV was titrated as described elsewhere [83]. Infectivity titers were determined using the JFH1 NS5A-specific mouse monoclonal antibody 9E10 (kindly provided by C. Rice, The Rockefeller University, New York, NY, USA) and expressed as the 50% tissue culture infective dose (TCID_50_)/mL.

### 2.8. Gel Electrophoresis and Western Blot Analysis

Denaturing SDS-polyacrylamide gel electrophoresis and Western blotting was performed as described elsewhere [84]. Dilutions of 1:4000 for DENV NS3 monoclonal antibody (GeneTex International Corporation, Hsinchu City, Taiwan), 1:1,000 for JFH1 NS5A monoclonal antibody (9E10 [83]), 1:1,000 for human phosphorylated AKT (P-AKT) rabbit monoclonal antibody (Ser473, Cell Signaling, Leiden, The Netherlands), 1:1000 for DDC rabbit polyclonal antibody (Affina Immuntechnik GmbH, Berlin, Germany; anti-DDC C-T [21]), which was raised against a peptide consisting of the last 22 C-terminal amino acids of the full-length human DDC, 1:500 for PI3K rabbit polyclonal antibody (Affina Immuntechnik GmbH, Berlin, Germany [21]), 1:8000 for GAPDH mouse monoclonal antibody (Santa Cruz Biotechnology, Dallas, TX, USA), 1:2000 for human lamin A rabbit polyclonal antibody [85], 1:2000 for calnexin rabbit polyclonal antibody (Sigma-Aldrich, Taufkirchen, Germany), and 1:6000 for pan-actin mouse monoclonal antibody (Merck-Millipore, Burlington, MA, USA), respectively, were used. A dilution of 1:2000 for the secondary anti-mouse and anti-rabbit horseradish peroxidase-conjugated antibodies (Cell Signalling, Leiden, The Netherlands) was used. Imaging quantification was performed by using Quantity I software (Bio-Rad, Hercules, CA, USA). Densitometry analysis data for the proteins of interest were normalized to the ones for actin.

### 2.9. Protein Immunoprecipitation under Non-Reducing Conditions

Co-immunoprecipitation of DDC from Huh7 cell lysate (input samples) was performed as described previously [21]. In brief, PI3K antibody was covalently immobilized on protein A beads with 20 mM dimethyl pimelimidate dihydrochloride (DMP) in 0.2 M triethanolamine pH 8.3. The antibody that had not been covalently bound to the beads was removed with the addition of 0.2 M glycine pH 2.5. Cells were lysed with RIPA buffer (20 mM Tris-HCl, pH 7.5, 150 mM NaCl, 1 mM EDTA, pH 8, 1% NP-40, and 1% sodium deoxycholate). The input samples were added to protein A beads without antibodies to remove proteins that bind non-specifically to the beads. The resulting supernatant was then added to protein A beads covalently bound to the antibody. The next day, the supernatant resulting from the immunoprecipitation, referred to as unbound lysate sample, was collected by centrifugation. Elution of the antigens (eluate) from the antibody-carrying beads was performed by raising the temperature (boiling). The antigens eluted were separated by SDS-PAGE and detected by immunoblotting.

### 2.10. Luciferase Assays

Firefly luciferase (F-Luc) activity in cell lysates was measured using a Luciferase Assay System (Promega Corporation, Madison, WI, USA), as recommended by the manufacturer. Renilla luciferase (R-Luc) activity in cell lysates was measured using 12 μM coelenterazine (Promega Corporation, Madison, WI, USA) in assay buffer (50 mM potassium phosphate, pH 7.4, 500 mM NaCl, and 1 mM EDTA). Measurements were taken in a GloMax 20/20 single-tube luminometer (Promega Corporation, Madison, WI, USA) for 10 s. Luciferase activities were normalized to the total protein amount determined using the Bradford assay reagent (Bio-Rad, Hercules, CA, USA).

### 2.11. Measurement of Intracellular ATP Levels

ATP was measured using the ViaLight HS BioAssay kit (Lonza, Basel, Switzerland) according to the manufacturer’s protocol in a GloMax 20/20 single-tube luminometer (Promega Corporation, Madison, WI, USA) for 1 s. ATP levels were normalized to total protein amounts.

### 2.12. Subcellular Fractionation

Subcellular fractionation of cytosolic, membrane, nuclear, and postnuclear fractions was performed by adapting the previously reported method [86]. In brief, 10^6^ Huh7 cells were seeded into 10-cm-diameter dishes the day before infection. Cells were infected with DENV at an MOI of 1 for 4 h at 37 °C. After 48 h, cells were washed three times with ice-cold PBS, trypsinized, and collected by centrifugation (700× *g*). Fractions were isolated as described previously [86] using different detergents. Specifically, we used digitonin to isolate the cytosolic fraction, Nonidet P-40 for the membrane fraction, 0,1% SDS for the nuclear fraction, and 1% SDS for the postnuclear fractions. Equal amounts of each fraction were subjected to SDS-PAGE followed by western blot analysis, as described above. Fraction purity was evaluated by detecting cytosolic (GAPDH), membrane (calnexin), and nuclear (lamin A/C) markers.

### 2.13. Indirect Immunofluorescence

Indirect immunofluorescence analysis of human DDC and PI3K in Huh7 cells was performed as following. Cells were seeded onto glass coverslips in 24-well plates at a density of 5 × 10^4^ cells per well. At 48 h post-infection, the cells were fixed with 500 μL of 3% paraformaldehyde for 10 min at room temperature and permeabilized by incubation in 500 μL of 0.5% Triton X-100 in PBS for 5 min. Staining of DDC was performed by using anti-DDC C-T polyclonal antibody at a dilution of 1:20, staining of DENV NS3 was performed by using a mouse monoclonal antibody (GeneTex International Corporation, Hsinchu City, Taiwan) at a dilution of 1:1000, and staining of JFH1 NS5A was performed by using a mouse monoclonal antibody (9E10 [83]) at a dilution of 1:1000 in PBS with 5% normal goat serum for 60 min. Bound primary antibodies were detected by using goat anti-Rabbit antibodies conjugated to Alexa-Fluor 488 or goat anti-mouse antibodies conjugated to Alexa-Fluor 546 at a dilution of 1:1000 in PBS containing 5% normal goat serum for 45 min in the dark. DNA was stained with TO-PRO-3 iodide (Thermo Fisher Scientific, Waltham, MA, USA) for 5 min. Finally, the cells were mounted on glass slides with Mowiol 4-88 (Sigma-Aldrich, Taufkirchen, Germany). Images were acquired with the TCS-SP confocal microscope (Leica Microsystems GmbH, Wetzlar, Germany). Fluorescence quantitation and colocalization analysis was carried out using Icy software (Institut Pasteur, Paris, France) [87,88]. Pearson’s correlation coefficient and Manders’ colocalization coefficients were calculated using Colocalization Studio plugin.

### 2.14. RNA Quantification by Reverse Transcription-Quantitative PCR (RT-qPCR)

Total cellular RNA was extracted using TRIzol reagent (Thermo Fisher Scientific, Waltham, MA, USA) according to the manufacturer’s instructions. cDNA synthesis was performed using Moloney murine leukemia virus reverse transcriptase (Promega Corporation, Madison, WI, USA) according to the manufacturer’s protocol and a mixture of the specific primers DV-A10940 (5′-ACCATTCCATTTTCTGGCGTT-3′) and YWHAZ-R for the DENV positive-strand RNA and the housekeeping gene 14-3-3-zeta polypeptide (YWHAZ) mRNA, respectively (3.5 pmol/μL of each primer), or oligo(dT) primers (New England Biolabs, Ipswich, MA, USA) for the DDC transcripts. Real-time quantitative PCR was performed using KAPA SYBR FAST qPCR Master Mix (Kapa Biosystems, Wilmington, MA, USA) as well as primer pairs specific for the DENV 3′UTR (DV-S10873: 5′-GAAAGACCAGAGATCCTGCTGTCT-3′ and DV-A10940), or the exons 10-12 of full-length DDC mRNAs (DDC-F: 5′-GAACAGACTTAACGGGAGCCTTT-3′ and DDC-R: 5′-AATGCCGGTAGTCAGTGATAAGC-3′). YWHAZ housekeeping gene was used as a normalization control in all reactions (YWHAZ-F: 5′-GCTGGTGATGACAAGAAAGG-3′ and YWHAZ-R: 5′-GGATGTGTTGGTTGCATTTCCT-3′), as it was confirmed that its expression was not affected upon viral infection or under low-oxygen conditions [89].

### 2.15. Chemicals

LY294002 (panPI3K inhibitor) was obtained from Cayman Chemical (Ann Arbor, MI, USA). Carbidopa, 5-hydroxytryptophan (5-HTP), l-dopa, Triton X-114, and protein A agarose beads were purchased from Sigma-Aldrich (Taufkirchen, Germany).

### 2.16. Statistical Analysis

In all diagrams, bars represent mean values of at least three independent experiments in triplicate or tetraplicate. Error bars represent standard deviation. Only results subjected to statistical analysis using Student’s *t*-test with *p* ≤ 0.05 were considered as statistically significant. Statistical calculations were carried out using Excel Microsoft Office^®^ (Microsoft Corporation, Redmond, WA, USA) or Prism (GraphPad Software, Inc., San Diego, CA, USA).

## 3. Results

### 3.1. Downregulation of l-Dopa Decarboxylase (DDC) by DENV and HCV Viruses

DDC, a cellular enzyme that exerts neurotransmitter biosynthetic function, has a wide distribution in a series of peripheral organs, including the liver, in which its biologic function is yet to be determined. Although DDC enzymatic products or their receptors have been reported to influence the entry of HCV and DENV [42,43,44,45], the implication of DDC in viral replication and pathogenesis has not been investigated. Here, we aimed to characterize the relationship of DDC with the *Flaviviridae* viruses HCV and DENV. For this, liver hepatoma Huh7 or Huh7.5 cells were used in infection studies, performed, in parallel, under two oxygen tensions: atmospheric (20% *v*/*v*), which is traditionally used in cell culture, and hypoxic (3% *v*/*v*), which is physiologically sensed by most hepatocytes in the liver and favors DENV and HCV RNA replication [68,69].

To investigate a putative regulation of DDC by DENV, Huh7 cells, preincubated at 20% or 3% (*v*/*v*) O_2_ for 18 h, were mock-infected or inoculated with DENV (DV-2, 16,681 strain) at a multiplicity of infection (MOI) of 1 for 4 h and, after medium exchange, further cultured for 24, 48, or 72 h at the conditions of preincubation. 

**Figure 1:**
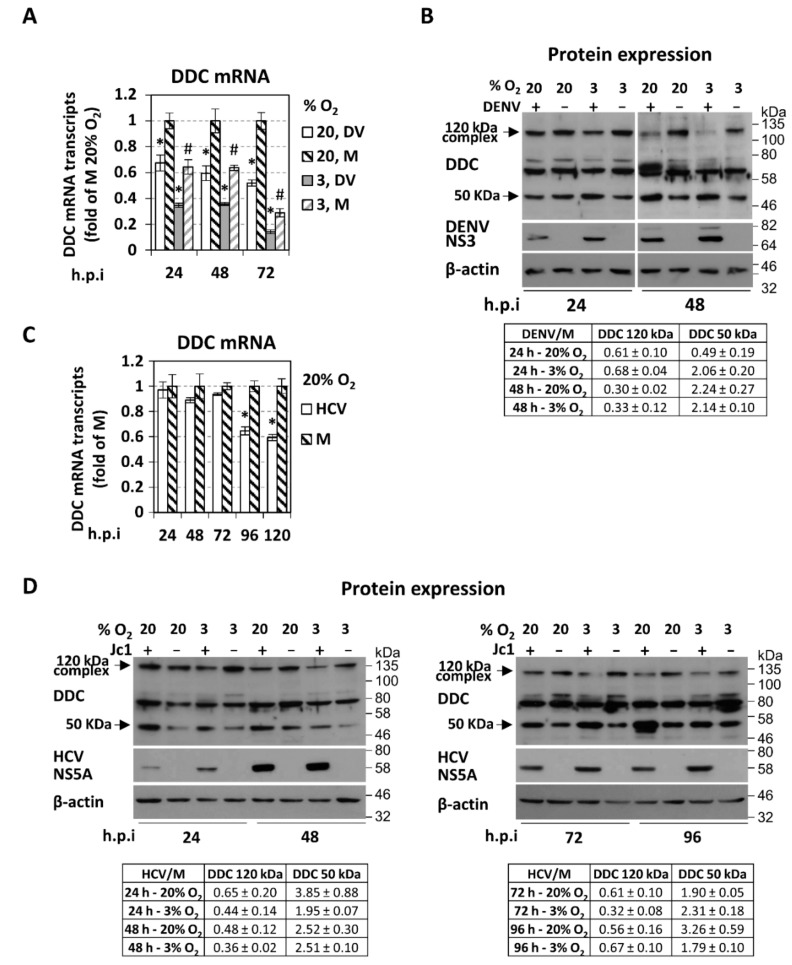
The impact of dengue (DENV) and hepatitis C (HCV) virus infection on the intracellular levels of l-dopa decarboxylase (DDC) mRNA and protein under 20% and 3% *v*/*v* O_2_. (**A**,**B**) Huh7 cells, preincubated at 20% or 3% *v*/*v* O_2_ for 18 h, were inoculated with cell-culture produced DENV virus particles (DV-2 16,681 strain, MOI = 1) for 4 h, washed twice with fresh culture medium, and further cultured for the indicated hours post-infection (p.i) under the respective oxygen condition. (**A**) Reverse transcription quantitative PCR (RT-qPCR) analysis of the intracellular total DDC mRNA levels from Huh7 DENV-infected cells. Values are expressed relative to the ones derived from mock-infected (M) cells under 20% O_2_ at each time-point. mRNA levels of the housekeeping gene 14-3-3-zeta polypeptide (YWHAZ) were used for normalization. Bars represent mean values from three independent experiments in triplicate. Error bars indicate standard deviations. * *p* < 0.001 vs. mock, # *p* < 0.001 vs. 20% O_2_. (**b**) (Top) Western blot analysis performed with (from top to bottom) anti-DDC-CT, anti-DENV NS3, or anti-β-actin antibodies. β-actin was used as loading control. Numbers on the right refer to the positions of molecular mass marker proteins. A representative experiment of three independent repetitions is shown. (Bottom) Densitometry analysis data for 120 kDa and 50 kDa DDC (mean values from three independent repetitions) were normalized to β-actin and to the values obtained with mock-infected cells. (**C**,**D**) Huh7.5 cells were inoculated for 4 h with cell-culture produced HCV virus (Jc1, MOI = 1) and further cultured for indicated h p.i under the respective oxygen condition. (**C**) RT-qPCR analysis of the total DDC mRNA levels from Huh7.5 Jc1-infected cells at 20% *v*/*v* O_2_. Values are expressed relative to the ones derived from mock-infected (M) cells at each time-point. YWHAZ mRNA levels were used for normalization. Error bars indicate standard deviations. * *p* < 0.001 vs. mock. (**D**) (Top) Western blot analysis of cells preincubated at 20% or 3% *v*/*v* O_2_ for 18 h before virus inoculation was performed using anti-DDC-CT, anti-HCV NS5A, or anti-β-actin antibodies. β-actin was used as loading control. A representative experiment of three independent repetitions is shown. (Bottom) Image quantification of DDC signals (mean values from three independent repetitions), normalized to β-actin and to the values obtained with mock-infected cells.

Total DDC mRNA from cell lysates was quantified using primers recognizing the vast majority of known DDC transcript variants. Moreover, as we have previously shown that in addition to the non-neuronal DDC transcript type, low but detectable levels of the neuronal DDC mRNA are also expressed in Huh7 cells [21], specific primers were used to discrete the viral effect on these two transcripts. As shown in Figure 1A, DENV infection reduced the level of total DDC mRNA (RT-qPCR) by up to 2-fold in both oxygen conditions, as observed by a kinetic analysis from 24 to 72 h post infection (h p.i), and in a virus titer-dependent manner (Figure S1A). Both non-neuronal and neuronal DDC transcript types were similarly decreased upon DENV infection (Figure S2A left). Additionally, 3% (*v*/*v*) O_2_ reduced *DDC* transcription in both DENV-infected and mock-infected cells (Figure 1A), in consistence with the downregulation of the majority of genes under hypoxia [90]. In the case of HCV infection in Huh7.5 cells that are highly permissive for this virus, a downregulation of DDC mRNA was detectable only at the later time-points of infection (Figure 1C), unlike the early effect of DENV.

To analyze the effect of DENV infection on DDC protein levels in the liver cell, we used the previously reported anti-DDC antibody raised against the last 22 C-terminal amino acids of the full-length human DDC (anti-DDC C-T) [21]. Consistent to our recent report [21], in Western blot analysis of Huh7 cells, the anti-DDC C-T antibody recognizes the ~50 kDa DDC monomer (Figure 1B) and a number of SDS-resistant DDC species, the most interesting of which exhibits a molecular weight of ~120 kDa, which is close to the hypothesized one for the catalytically active, dimeric form of the protein [18]. DENV, at 24 h p.i, had a negative effect on the levels of both the 50 kDa monomer and the 120 kDa complex of DDC, as compared to the mock-infected (M) cells incubated at 20% O_2_. Moreover, at 48 h p.i, the virus decreased the levels of the 120 kDa complex, concomitant with an accumulation of the 50 kDa monomer (Figure 1B), in a virus titer-dependent manner (Figure S1 right). These observations suggest a negative regulation of DDC by DENV infection. Interestingly, under 3% O_2_ tension, the accumulation of the 50 kDa DDC was already detectable at 24 h p.i. This is possibly due to the increased DENV replication under this condition [69], as confirmed by the detected levels of DENV NS3 protein (Figure 1B). On the other hand, in mock-infected cells, no significant alteration in DDC protein levels was observed between the two oxygen conditions, in contrast to DDC mRNA.

Similarly, HCV also exerted a negative effect on the 120 kDa DDC complex with a concomitant accumulation of 50 kDa monomer, with similar kinetics in the two oxygen conditions (Figure 1D), as shown upon infection of Huh7.5 cells with the Jc1 chimeric strain (MOI = 1). This was detected already at 24 h p.i even at 20% O_2_.

On the other hand, viral infection does not seem to affect the levels of the other SDS-resistant DDC immunoreactive species (Figure 1B,D), with molecular weights between 60 and 80 kDa. These species were also previously shown in [21]. Based on the accumulating data for complex splicing mechanisms and alternative exons, resulting in multiple DDC transcript variants [26,34,35], these DDC forms possibly represent products of not yet characterized alternatively spliced DDC mRNA variants, post-translationally modified DDC isoforms, or SDS-resistant protein complexes of DDC.

**Figure 2:**
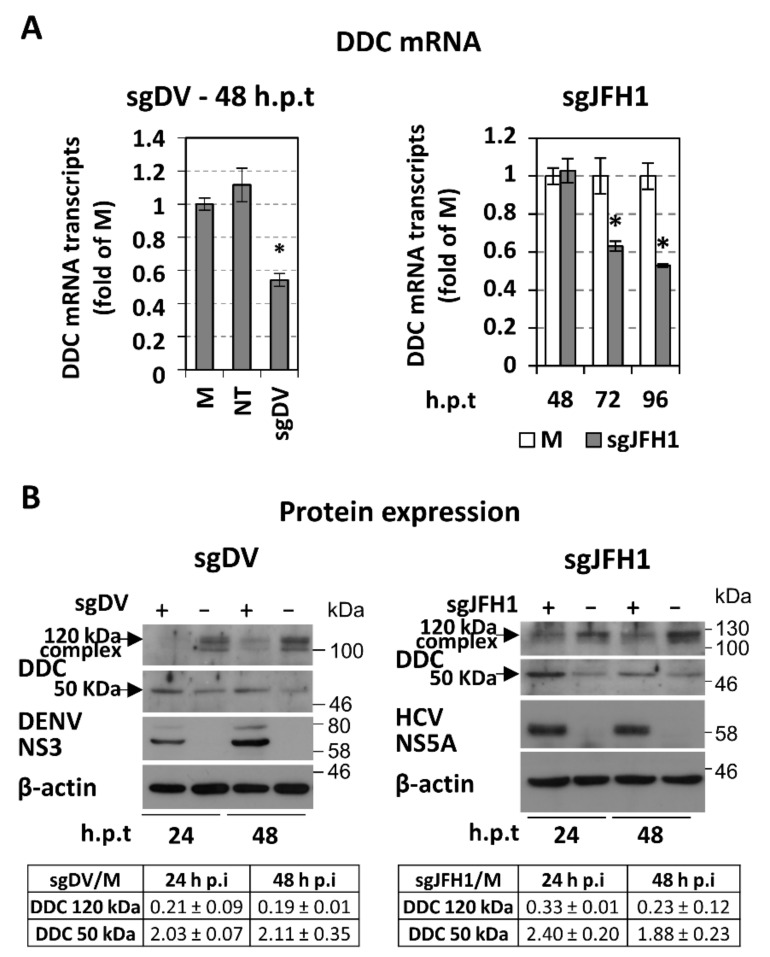
The effect of DENV and HCV genome replication on the intracellular levels of DDC mRNA and protein. (**A**,**B**) Huh7 and Huh7-Lunet cells were electroporated with in vitro transcribed subgenomic reporter DENV-2 16,681 RNA (sgDV; left) or HCV JFH1 RNA (sgJFH1; right), respectively (10 μg RNA/4 × 10^6^ cells), and further cultured for the indicated h p.t under 20% O_2_. Non-transfected (NT) cells or cells mock-electroporated with a capped-polyadenylated *Renilla* luciferase expressing RNA (M) were used as controls. (**A**) RT-qPCR analysis of the total DDC mRNA levels of DENV-replicon (left) and HCV-replicon (right) transfected cells. Values are expressed relative to the ones derived from non-transfected (NT) cells or mock-transfected (M) cells, at each time-point. YWHAZ mRNA levels were used for normalization. Bars represent mean values from three independent experiments in triplicate. Error bars indicate standard deviations. * *p* < 0.001 vs. mock-transfected cells. (**B**) Western blot analysis using anti-DDC-CT, anti-DENV NS3 (left), or anti-HCV NS5A (right) and anti-β-actin antibodies. β-actin was used as loading control. A representative experiment of three independent repetitions is shown. (Bottom) Image quantification of DDC signals (mean values from three independent repetitions), normalized to β-actin and to the values obtained with mock-transfected cells.

To examine whether the regulation of DDC by DENV and HCV is dependent on the viral RNA replication stage, Huh7 and Huh7-Lunet cells were electroporated with a subgenomic reporter replicon of DENV-2 16,681 or HCV JFH1, respectively, and further cultured for different hours post-transfection (p.t). Both DDC mRNA (Figure 2A) and protein (Figure 2B) levels were similarly affected as upon viral infection. These results were confirmed by electroporation of Huh7-Lunet cells with the Jc1 full-length RNA (Figure S3).

Thus, we provide evidence for a conserved negative, viral genome replication-related effect from both *Flaviviridae* viruses DENV and HCV on the levels of DDC mRNA and the 120 kDa putatively functional DDC complex, concomitant with an accumulation of the 50 kDa DDC monomer, but with different kinetics between the two viruses. Moreover, these virus-mediated effects on DDC were validated in a more biologically relevant oxygen condition.

### 3.2. Effect of DENV-/HCV-Infection on DDC Subcellular Localization and DDC-PI3K Interaction

**Figure 3:**
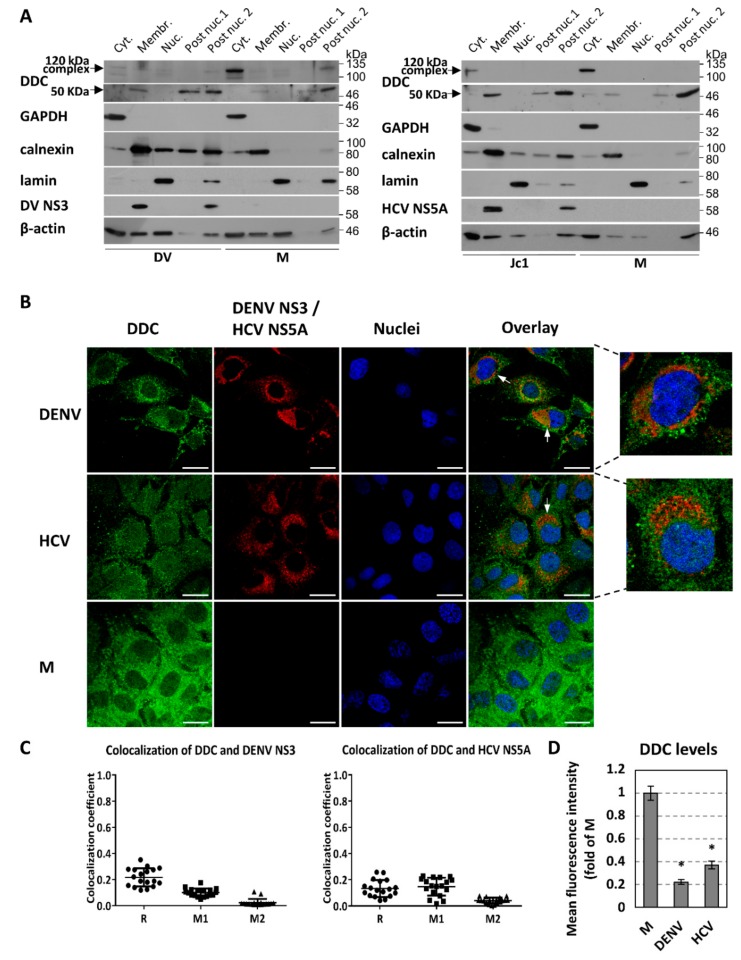
The influence of DENV and HCV infection on the subcellular fractionation and localization of DDC protein. (**A**) Subcellular distribution of DDC protein as detected by fractionation of Huh7 cells infected with DENV (DV-2 16681, MOI = 1) for 48 h (left), Huh7.5 cells infected with HCV (Jc1, MOI = 1) for 72 h (right) and mock-infected cells. Intracellular distribution of DDC protein in soluble cytosolic (Cyt), membrane-associated (Membr), nuclear (Nuc), and postnuclear insoluble (Post-Nuc) fractions was evaluated by Western blot analysis. GAPDH, calnexin, and lamin A served as specificity markers for the various fractions. The distribution of DENV NS3 and HCV NS5A were also analyzed. β-actin was used as loading control of infected and mock-infected cells. A representative experiment of three independent repetitions is shown for each virus. (**B**–**D**) The subcellular localization of DDC, as detected by immunofluorescence, in relation to viral proteins in Huh7.5 cells infected with DENV (MOI = 1) for 48 h (upper panel) or Jc1 (MOI = 1) for 72 h (middle panel) or mock-infected cells (lower panel). (**B**) Immunofluorescence analysis of DDC (green) using anti-DDC C-T antibody, followed by confocal microscopy. DENV NS3 or HCV NS5A proteins were also stained with specific antibodies (red). Nuclei were stained with TO-PRO-3 iodide (blue). On the right, merged images of the green, red, and blue fluorescence are shown. Bar, 20 μΜ. White arrows indicate areas in the infected cells with high viral protein levels where DDC is absent. At the far right, magnifications of single cell images are presented. (**C**) Colocalization coefficients between DDC and viral proteins. Graphical representation of the Pearson correlation coefficient (R) and Manders’ colocalization coefficient (M1, M2) values between DDC and DENV NS3 (left) or HCV NS5A (right). Each spot represents a single analyzed infected cell. (**D**) Fold difference of mean DDC fluorescence intensity per cell, between DENV- or HCV-infected and mock-infected cells. Values are expressed relative to the ones derived from mock-infected (M) cells. Bars represent mean values obtained from three experiments (~30 analyzed cells/experiment). Error bars indicate standard deviations. * *p* < 0.001 vs. mock-infected cells.

Several lines of evidence have shown that DDC is localized both in the cytoplasm and in the cellular membrane fractions as an integral or membrane-associated protein, as well as that the membrane-bound protein is released through an enzyme-dependent mechanism [91,92,93,94]. To examine if viral infection influences the subcellular distribution of the different DDC species in hepatocytes, we performed cell fractionation using a method that allows separation of cytosolic, membrane, nuclear, and insoluble (of post-nuclear fractions) proteins [86]. For this, we used lysates from Huh7 cells infected with DENV-2 16681, Huh7.5 cells infected with HCV Jc1, or mock-infected cells. As shown in Figure 3A, the 120 kDa DDC complex was detected in the cytosolic fraction, whereas the 50 kDa monomer was accumulated in the membranous and the insoluble fractions. The former was almost vanished in the infected cells, in accordance with our results from the western blot analysis of crude cell extracts. Thus, viral infection reduces the levels of cytoplasmic 120 kDa DDC and increases the membrane-associated 50 kDa DDC, while it does not change the distribution of the two DDC species in the individual cell fractions.

Similarly, viral infection does not influence the biochemical properties of DDC protein, and more specifically, the hydrophobicity of DDC species, as shown in DENV- or mock-infected Huh7 cells subjected to phase separation assay using Triton X-114 and western blot analysis (Figure S4). Moreover, the 50 kDa DDC monomer is detected in the detergent insoluble (phospholipid-enriched) fraction, consistently with our previous findings [92,93,94], which shows high hydrophobicity. The 120 kDa complex was not detectable, possibly due to disruption by Triton X-114.

Next, we examined the localization of DDC relative to DENV and HCV replication sites, by immunofluorescence analysis in infected Huh7.5 cells, as compared to mock-infected ones. We observed that DDC did not colocalize significantly with DENV NS3 (Pearson correlation coefficient: R = 0.21 ± 0.07, Manders’ colocalization coefficient: M1 = 0.10 ± 0.03) or HCV NS5A (R = 0.13 ± 0.07, M1 = 0.15 ± 0.07) (Figure 3B,C), despite that a significant portion of DDC was detected in the membrane fraction of the cell (Figure 3A). Thus, DDC appears to be absent from the viral replication sites, which is emphasized by white arrows in Figure 3B. As a control, we verified the colocalization of DENV NS3 protein with the endoplasmic reticulum (ER) marker calnexin (Figure S5, R = 0.68 ± 0.14, M1 = 0.46 ± 0.09), as well as the DENV-induced enhancement of ER-staining [95,96]. Interestingly, in both DENV- and Jc1-infected cells, we observed overall lower DDC amounts, as compared to mock-infected cells (Figure 3B,D), which correlates well with the virus-mediated downregulation of DDC mRNA and 120 kDa DDC complex (Figure 1). Moreover, in DENV-infected cells, DDC appears to be accumulated in bright cytoplasmic spots, which is not the case for HCV.

**Figure 4:**
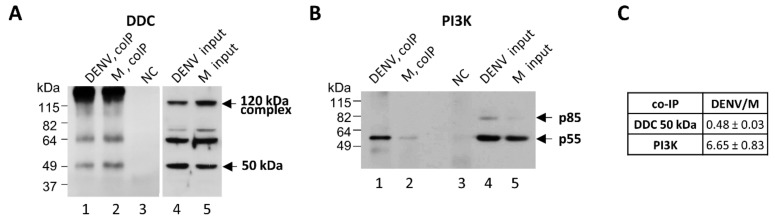
The influence of DENV infection on the DDC-PI3K complex. (**A**–**C**) Whole lysates of Huh7 DENV-infected cells (DV-2 16681, MOI = 1) at 48 h p.i were used in immunoprecipitation experiments, performed with anti-PI3K antibody. Western blot analysis was performed with anti-DDC-CT (**A**) or anti-PI3K (**B**) antibodies. Lysates from mock-infected cells were used as control. Lanes 1,2: Eluates of the co-immunoprecipitation (co-IP). Lane 3: Eluate of co-IP without lysate, used as a negative control (NC). Lanes 4, 5: Input cell lysates before co-IP. (**C**) Image quantification of co-immunoprecipitated DDC and PI3K signals (mean values from three independent repetitions), normalized to the values obtained with mock-transfected cells.

Next, we sought to investigate a putative effect of DENV on the physical interaction of DDC with PI3K, which we have recently shown for the first time [21]. For this, we employed a DDC-PI3K co-immunoprecipitation assay previously validated in Huh7 cells [21]. Cells were infected with DENV-2 16,681 or mock-infected. At 48 h p.i, a previously characterized anti-PI3K polyclonal antibody [21] was used to immunoprecipitate the DDC-PI3K complex from cell lysates, followed by the detection of DDC, in western blot analysis, using the anti-DDC C-T polyclonal antibody. In consistence to our previous report [21], the membrane-bound 50 kDa DDC monomer and the ~60 kDa species were detected in the western blot analysis of the immunoprecipitates (Figure 4A, lanes 1,2), while the binding of PI3K p55 subunit on the beads–antibody conjugate was also confirmed (Figure 4B, lanes 1,2). Interestingly, DENV-infection resulted in a decrease of the amount of DDC found in complex with PI3K (Figure 4A, lanes 1,2), although it increased the intracellular levels of the 50 kDa DDC, as confirmed by western blot analysis in cell lysates (Figure 4A, lanes 4,5). Concomitantly, DENV reduced the binding of PI3K to the antibody-coated beads (Figure 4B, lanes 1,2), which may be indicative of a conformational change of PI3K upon binding to DDC.

### 3.3. Effect of Overexpression and Chemical Inhibition of DDC on Viral Replication and Infectivity

**Figure 5:**
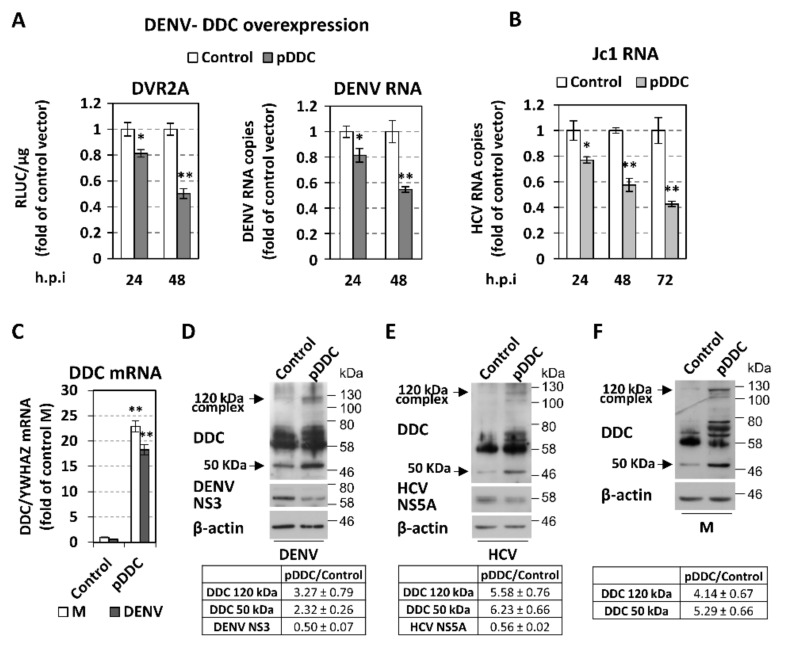
Effect of DDC overexpression on DENV replication. Huh7 or Huh7.5 cells were electroporated with pcDNA3.1(+)-DDC (pDDC) or the control vector, 24 h p.t were inoculated with DV-2 16,681 reporter (DVR2A, MOI = 0.1) or non-reporter (DENV, MOI = 1) virus or HCV Jc1 (MOI = 1) for 4 h, and were lysed at the indicated h p.i. (**A**) Left: DVR2A replication-derived Renilla luciferase (R-Luc) activity was quantified by chemiluminescence-based assay and expressed as relative light units (RLU) per μg of total protein amount. Right: DENV positive-strand RNA levels were quantified by RT-qPCR and YWHAZ mRNA was used for normalization. (**B**) Jc1 positive-strand RNA levels were quantified by RT-qPCR and normalized. Bars represent mean values from three independent experiments in triplicate. Error bars indicate standard deviations. * *p* < 0.01 vs. control, ** *p* < 0.001 vs. control. (**C**–**F**) Total DDC mRNA and protein levels in cells electroporated with the pDDC plasmid or the control vector and then DENV-, HCV-, or mock-infected (M) for 48 h. (**C**) DDC mRNA levels were determined by RT-qPCR and normalized to YWHAZ mRNA. Bars represent mean values from three independent experiments in triplicate. Error bars indicate standard deviations. ** *p* < 0.001 vs. control vector-transfected cells. (**D**–**F**) (Top) Western blot analysis was performed in the electroporated cells infected with DENV (**D**), HCV (**E**), or mock-infected (**F**), using anti-DDC-CT, anti-DENV NS3, or anti-HCV NS5A and anti-β-actin antibodies. β-actin was used as loading control. A representative experiment of three independent repetitions is shown. (Bottom) Image quantification of DDC and DENV NS3 or HCV NS5A signals (mean values from three independent repetitions), normalized to β-actin and to the values obtained with control vector-transfected cells.

Having identified DDC as a new host factor that is regulated by DENV and HCV infection, we sought to identify the influence of DDC on viral proliferation. Therefore, we first examined the effect of DDC overexpression on DENV replication. To upregulate DDC intracellular levels, Huh7 cells were mock-electroporated or electroporated with a mammalian plasmid vector that expresses DDC [33,77] under the transcriptional control of the cytomegalovirus (CMV) promoter (pcDNA3.1(+)-DDC). Cells, 24 h post-electroporation, were infected with HCV Jc1, DENV-2 16681, or a reporter-DENV virus (DVR2A) that expresses *Renilla* luciferase. The levels of HCV and DENV RNA or DENV replication-derived Renilla luciferase activity were determined in cell lysates at the indicated h p.i. As shown, the overexpression of DDC affected negatively the replication of DENV (Figure 5A) and HCV (Figure 5B). Consistently, a significant reduction was observed at the level of viral protein expression upon DDC overexpression (Figure 5D,E). The overexpression of DDC mRNA (Figure C) and protein (Figure 5D–F) as compared to mock-electroporated cells, was confirmed. Concerning DDC protein, Figure 5D–F shows the concomitant overexpression of the ~50 kDa monomer and ~120 kDa form, as well as of the other SDS-resistant DDC immunoreactive species of 60–80 kDa, which confirms their DDC specificity.

**Figure 6:**
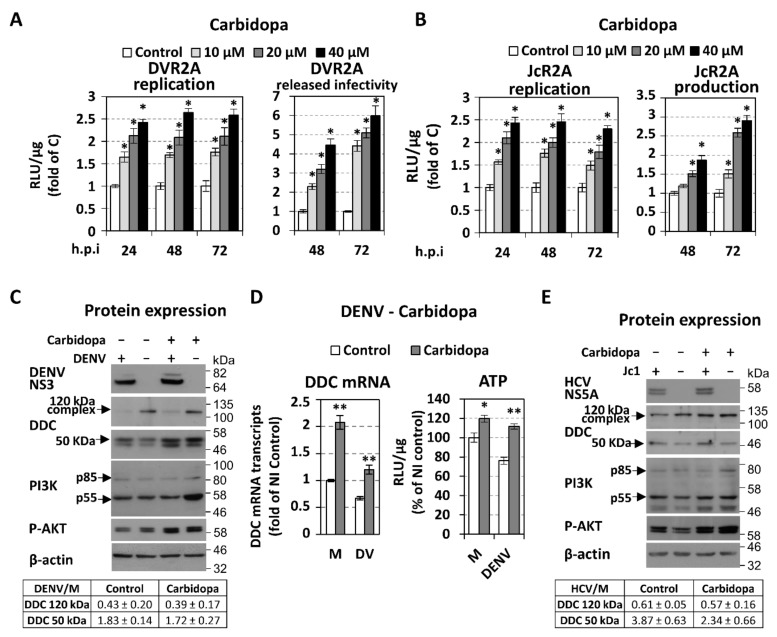
Effect of the DDC inhibitor carbidopa on DENV and HCV replication and released infectivity. (**A**,**B**) Huh7 cells inoculated with DVR2A (MOI = 0.1) and Huh7.5 cells inoculated with JcR2A (MOI = 0.5) for 4 h, were treated, or not (control), with different concentrations of carbidopa for the indicated h p.i. Cells were then lysed and virus replication–derived R-Luc activity was determined (left). Naïve cells were inoculated for 4 h with supernatant from the infected cells treated or not with carbidopa for 48 or 72 h. Cells were lysed at 72 h p.i and R-Luc activity, indicative of the released infectivity levels of the first round of infection, was determined (right). Luciferase levels are expressed as RLU/μg of total protein amount. Values from control cells were set to one for each time point. Bars represent mean values from at least three independent experiments in triplicate. * *p* < 0.001 vs. control (**C**,**E**) Huh7 cells inoculated with DENV (DV-2 16681, MOI = 1) or Huh7.5 cells inoculated with HCV (Jc1, MOI = 1) for 4 h were treated with carbidopa (20 μM) and further cultured for 48 h p.i. (Top) Western blot analysis was performed in cell lysates using anti-DENV NS3 (B) or HCV NS5A (E), anti-DDC-CT, anti-PI3K, anti-P-AKT (phosphorylated AKT), and anti-β-actin antibodies. β-actin was used as loading control. Representative experiments of three independent repetitions are shown. (Bottom) Image quantification of DDC signals (mean values from three independent repetitions), normalized to β-actin and to the values obtained with mock-infected cells. (**D**) Effect of the DDC inhibitor carbidopa (20 μM) on DDC mRNA (left) and on intracellular ATP levels (right) of Huh7 cells inoculated for 4 h with DENV (DV-2 16681, MOI = 1) or mock-infected (M) and further cultured for 48 h. DDC mRNA was quantified by RT-qPCR and YWHAZ mRNA was used for normalization. ATP was measured using a chemiluminescence-based assay and expressed as RLU/μg of total protein amount. Values obtained from the control non-treated and mock-infected cells were set to 1 or 100%. Bars represent mean values from two independent experiments performed in triplicate. Error bars indicate standard deviations. * *p* < 0.05 vs. control, ** *p* < 0.001 vs. control.

Next, we examined the effect of DDC enzymatic activity, on DENV and HCV proliferation, using its competitive inhibitor, carbidopa [97], which irreversibly binds and deactivates the DDC cofactor PLP [98,99]. For this, we used the *Renilla* luciferase reporter viruses DVR2A and HCV JcR2A. Infected cells were treated with different concentrations of carbidopa for 24–72 h and viral replication-derived luciferase activity was determined. In addition, the effect of carbidopa on virus released infectivity was determined by infecting naïve Huh7 or Huh7.5 cells with supernatants of carbidopa-treated infected cells. As shown in Figure 6A,B (see also Figure S6; replication kinetics of the reporter viruses), carbidopa significantly enhanced the replication of both viruses up to 2.5-fold (left panels), as well as the released infectivity up to 6-fold for DENV and 3-fold for HCV (right panels). In agreement, enhanced accumulation of DENV NS3 and HCV NS5A proteins was detected in DENV- or HCV Jc1-infected cells treated with carbidopa (Figure 6C,E). Carbidopa affected at least the viral RNA replication step, as verified upon cell electroporation with the subgenomic replicons of DENV or HCV (Figure S7A). Furthermore, treatment of infected cells with the DDC substrates l-dopa and 5-hydroxytryptophan (5-HTP), in non-cytotoxic concentrations, reduced viral replication (Figure S7B). l-dopa and 5-HTP are the immediate precursors of dopamine and serotonin, respectively, and are known to raise the concentration of these DDC products [16,100]. The above data indicate that the enzymatic activity of DDC negatively correlates with the replication of both *Flaviviridae* viruses.

As mentioned above, in contrast to HCV, the effect of carbidopa on DENV-released infectivity was more pronounced than the one observed at the replication level. The difference in the released infectivity between the two viruses may be related to an interesting effect of carbidopa on cell viability. Indeed, carbidopa ameliorated DENV-induced cell death at 48 h p.i (Figure 6D right) as determined by measuring intracellular ATP content [101], whereas slightly increased cell viability in mock-infected (in consistence with our previous results [21]) and HCV-infected cells (data not shown). This ATP upregulation coincided well with the elevated levels of AKT phosphorylation, observed upon carbidopa treatment, in both infected and mock-infected cells (Figure 6C,E). Interestingly, carbidopa affected positively the expression of PI3K regulatory subunits in mock-infected cells in agreement with our previous results [21].

Western blot analysis also revealed that, as in the case of other DDC inhibitors [102] and in agreement with our previous study [21], carbidopa treatment caused an accumulation of DDC as observed by the enhanced levels of DDC mRNA (Figure 6D) and of both 120 kDa and 50 kDa DDC protein forms (Figure 6C,E). However, carbidopa did not affect the virus-mediated regulation of the DDC protein (Figure 6C,E).

In total, we provide evidence for a bidirectional relationship between DDC and viral infection, which may be important for viral replication establishment and pathogenesis.

### 3.4. The Role of PI3K in DENV- and HCV-Mediated DDC Regulation

**Figure 7:**
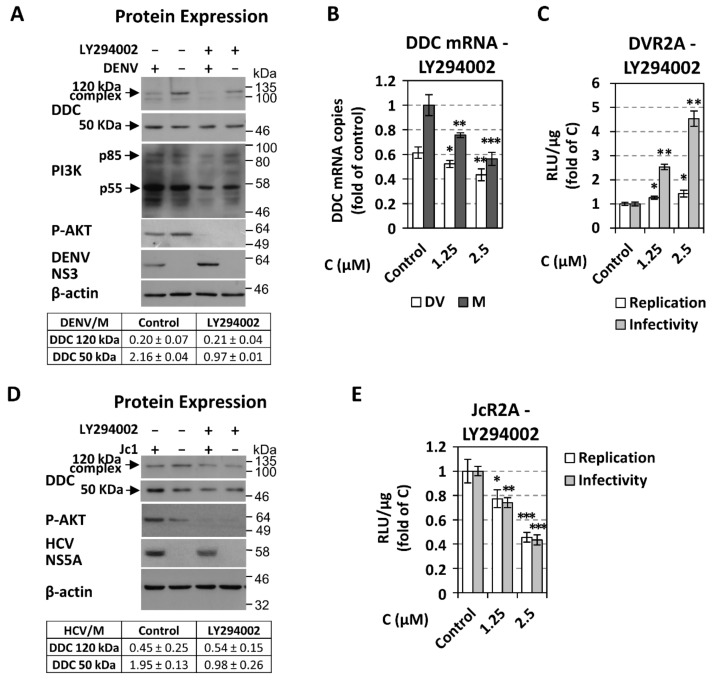
Effect of PI3K inhibition on DENV- and HCV-mediated DDC protein regulation in infected cells. (**A**–**C**) Huh7 cells inoculated with DV-2 16,681 non-reporter (DENV, MOI = 1) or reporter (DVR2A, MOI = 0.1) virus for 4 h, were treated with different concentrations of the PI3K inhibitor LY294002 for 48 h, respectively. (**A**) (Top) Western blot analysis of lysates of DENV- or mock-infected cells, treated or not with 2.5 μM LY294002, using anti-DDC-CT, anti-PI3K, anti-P-AKT, anti-DENV NS3, or anti-β-actin antibodies. β-actin was used as loading control. Representative experiments of three independent repetitions are shown. (Bottom) Image quantification of DDC signals (mean values from three independent repetitions), normalized to β-actin and to the values obtained with mock-infected cells. (**B**) RT-qPCR analysis of DDC mRNA levels, in DENV- or mock-infected cells, treated, or not with LY294002 at the specified concentrations. YWHAZ mRNA was used for normalization. (**C**) As control experiment, viral RNA replication in DVR2A-infected cells treated with LY294002 was determined by measurement of virus-derived R-Luc activity levels. Cells treated with the solvent DMSO were used as control. For the determination of released infectivity, naïve cells were inoculated for 4 h with supernatant of infected cells treated with LY294002 or DMSO. Then, cells were lysed at 72 h p.i and R-Luc activity was determined. * *p* < 0.05 vs. control, ** *p* < 0.01 vs. control, *** *p* < 0.001 vs. control. (**D**,**E**) Huh7.5 cells inoculated with Jc1 or JcR2A (MOI = 1) for 4 h were treated with different concentrations of LY294002 for 72 h. (**D**) (Top) Western blot analysis of lysates of HCV- or mock-infected cells, treated or not with 2.5 μM LY294002, using anti-DDC-CT, anti-P-AKT, anti-HCV NS5A, or anti-β-actin antibodies. β-actin was used as loading control. Representative experiments of three independent repetitions are shown. (Bottom) Image quantification of DDC signals (mean values from three independent repetitions), normalized to β-actin and to the values obtained with mock-infected cells. (**E**) Effect of LY294002 on JcR2A replication and released infectivity in infected Huh7.5 cells using the same experimental setup described for DVR2A in C. * *p* < 0.05 vs. control, ** *p* < 0.01 vs. control, *** *p* < 0.001 vs. control.

Based on DDC-PI3K interaction, we sought to investigate if PI3K inhibition has any putative implication on virus-mediated regulation of DDC. For this, DENV-infected Huh7 and HCV Jc1 strain-infected Huh7.5 cells were treated with different concentrations of PI3K inhibitor LY294002, which has been shown not to affect the DDC-PI3K complex formation [21]. Interestingly, the inhibitor reverted the virus-mediated effect on the levels of 50 kDa DDC monomer (Figure 7A,D) and DDC mRNA (Figure 7B). The effect of PI3K inhibition on virus-DDC relationship appeared to be independent from its effect on viral replication, as LY294002 differentially influenced DENV and HCV replication, shown by viral protein levels (Figure 7A,D) and virus-derived luciferase activity (Figure 7C,E) in consistence with previous reports [59,65,66,67]. In parallel, LY294002 downregulated DDC mRNA (Figure 7B) and protein (Figure 7A,D). As expected [103], LY294002, in non-cytotoxic concentrations (data not shown), significantly decreased the amount of PI3K subunits and AKT phosphorylation (Figure 7A,D). In addition, we confirmed the downregulation of P-AKT at a late time-point of DENV infection (48 h, Figure 7A) [62] and its upregulation upon HCV infection (Figure 7D) [67] as compared to mock-infected cells.

To further characterize the implication of PI3K on virus-DDC interaction, we sought to investigate the role of its downstream target AKT in this relationship, by using AKT inhibitor VIII. AKT inhibition, similarly to PI3K inhibitor, abrogated the DENV- and HCV-mediated effect on the levels of the 50 kDa DDC monomer but not on the 120 kDa complex (Figure S8A,C). Moreover, in mock-infected cells, AKT inhibitor, in contrast to PI3K inhibitor, did not alter DDC protein and mRNA levels (Figure S8B).

In total, our data revealed the importance of PI3K/AKT pathway on virus-dependent DDC regulation, suggesting that PI3K/AKT pathway should be active, so that *Flaviviridae* viruses can affect DDC.

### 3.5. Inverse Correlation Between DDC mRNA and HCV Replication In Vivo

**Figure 8:**
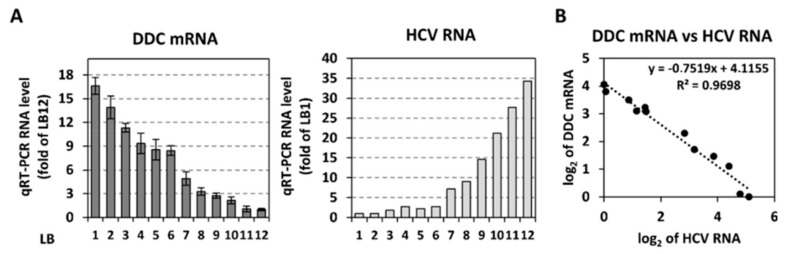
Inverse correlation between HCV RNA and *DDC* gene expression levels in human liver biopsies. (**A**) Total RNA was isolated from 12 liver samples (LB 1 to 12) from patients with chronic hepatitis C previously characterized for their HCV RNA amounts (right) with the branched DNA assay (Figure adapted from Vassilaki et al. 2013 [68], Copyright ^©^ American Society for Microbiology). The DDC mRNA in these samples was quantified by RT-qPCR (left). YWHAZ mRNA levels were used for normalization. Values are expressed relative to those obtained with sample LB 12. Bars represent mean values of DDC RNA copies from three technical replicates, and error bars indicate standard deviations. (**B**) XY scatter plot and linear regression analysis of log 2-transformed relative levels of DDC mRNA versus HCV genome. R^2^ indicates coefficient of determination.

To examine if there is a relationship between the replication levels of a *Flaviviridae* virus and the expression of *DDC* in vivo, we quantified by RT-qPCR the DDC mRNA levels in 12 liver fine-needle biopsy specimens from patients with liver fibrosis due to chronic HCV infection, that have been previously characterized for their HCV RNA quantity, as well as the expression profile of PI3K/AKT target genes [68]. As shown in Figure 8A, liver biopsy (LB) samples 1 to 6, while containing the lower HCV RNA amounts (right), contained the higher DDC mRNA(s) levels (left). Moreover, LB samples 7 to 12, showing the higher HCV RNA amounts, contained the lower levels of DDC transcripts. As indicated by the scatter plot and the coefficient of determination in Figure 8B, a strong correlation was determined (R^2^ = 0.97). Despite the limited number of clinical samples, these results argue for an in vivo inverse correlation between *DDC* expression and HCV replication.

Thus, our observations from the in vitro and in vivo conditions highlight the importance of DDC as a potential novel determinant of virus replication and virus–host interaction.

## 4. Discussion

**Figure 9:**
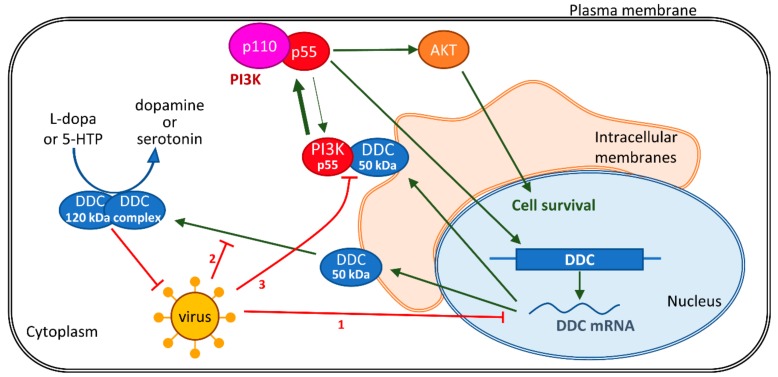
Schematic representation of the proposed mechanism concerning the relationship between viral infection, DDC, and PI3K/AKT pathway. DENV or HCV infection downregulates DDC at three different levels: (1) downregulates DDC mRNA, (2) reduces the release of the 50 kDa DDC monomer from intracellular membranes and subsequent formation of the 120 kDa dimeric complex, possibly representing the catalytically active form of DDC, and (3) disturbs the binding between the 50 kDa DDC monomer and PI3K p55 subunit, which releases PI3K p55 to interact with PI3K p110 and increases the activity of the PI3K/AKT pathway in favor of cell survival and viral replication during all stages of HCV infection and at the early phase of DENV infection. In turn, the enzymatic activity of DDC downregulates DENV and HCV propagation.

In the present study, we reveal that l-dopa decarboxylase (DDC), an enzyme that catalyzes the biosynthesis of dopamine, is a determinant factor of the *Flaviviridae* viruses DENV and HCV life cycle and, in turn, is negatively regulated by viral infection. Specifically, both DDC mRNA and protein are inversely correlated with the viral genome replication levels in hepatocytes, as indicated by infection and replicon transfection studies, as well as, in the case of DDC mRNA, by the use of liver biopsies of chronic HCV patients. Furthermore, DDC overexpression or enzymatic inhibition reduced or enhanced, respectively, DENV and HCV viral propagation. Finally, based on our previous evidence of DDC interaction with PI3K [21], as well as the known role of PI3K in virus replication, we investigated the implication of PI3K on the association of HCV and DENV with DDC. Our results are discussed in detail below, and the proposed mechanism of the tripartite relationship between virus, DDC, and PI3K is depicted in Figure 9.

A negative effect was observed on *DDC* gene expression upon HCV and DENV infection in Huh7-derived cells. In the case of DENV, this downregulation was already apparent at 24 h p.i (Figure 1A,C), under atmospheric oxygen (20% *v*/*v* O_2_), used conventionally in tissue culture, as well as in conditions that mimic liver hypoxia (3% *v*/*v* O_2_) and favor viral RNA replication [68,69]. However, the effect of HCV infection on DDC mRNA was detectable only at late time-points of infection (96 h). This may be related to the differences in the replication cycles of these viruses.

Concerning the effect of HCV and DENV on DDC protein, our results showed that viral infection reduces the levels of an ~120 kDa DDC species, with a concomitant accumulation of the 50 kDa DDC monomer (Figure 1) [18]. The ~120 kDa DDC has a molecular weight close to the hypothesized one for the catalytically active form of the protein [18] and possibly represents a DDC SDS-resistant hydrophobic complex, as supported by its overexpression upon DDC cDNA transfection (Figure 5) and its disassociation after TritonX-114 phase separation (Figure S4). As an exception, DENV initially reduced the 50 kDa DDC monomer at 20% O_2_, which could be attributed to the early downregulation of the DDC gene referred to above. At 3% O_2_, the 50 kDa monomer has already accumulated at 24 h p.i., possibly due to the enhanced viral RNA replication under hypoxia (first shown in [69]). In the case of HCV, no initial reduction of the 50 kDa DDC monomer was observed, which is consistent with the late downregulation of *DDC* gene expression by this virus. Taking into account that the virus-mediated effects on the DDC protein and mRNA occur with different kinetics, especially in the case of HCV infection, where DDC protein is affected much earlier than the mRNA, we hypothesize that viruses may utilize distinct regulatory mechanisms for DDC. This is not surprising, as DDC expression and function is under the control of complicated and mostly uncharacterized mechanisms [19]. Virus-mediated DDC regulation is dependent, at least in part, on the viral RNA replication stage (Figure 2). Moreover, the slightly smaller than 50 kDa DDC species, which is detected occasionally in our experiments (Figure 6C and Figure S8C) and may be produced by the mRNA variant lacking exon 3 (Figure S2B, [34]), has a similar virus-mediated regulation profile as the 50 kDa DDC monomer. On the other hand, viral infection does not seem to affect the levels of other DDC immunoreactive species between 60 and 80 kDa (Figure 1), so they were not further studied.

Moreover, our results showed that viral infection reduces the levels of cytoplasmic 120 kDa DDC and increases the membrane-associated 50 kDa DDC, while it does not change their distribution in the individual cell fractions (cytosolic, membrane, nuclear and insoluble/post-nuclear). Interestingly, the detection of the 50 kDa monomer in the membranous and the insoluble fractions (Figure 3A), is in agreement with its high hydrophobicity shown here and in previous reports for neuronal tissue, by TritonX-114 phase separation assay (Figure S4) [94]. In contrast, the detection of the 120 kDa DDC complex in the cytosolic fraction coincides with the reported localization of the enzymatic functional form of DDC [91]. Based on these results and on previous observations that the 50 kDa DDC is released from the membrane fraction in an enzyme-dependent mechanism [92,93,94], DENV and HCV could downregulate DDC through inhibition of its release from the membranes to the cytoplasm.

In consistence with the results on DDC mRNA, DENV- and HCV-infected cells contain significantly lower total intracellular DDC protein amounts, as compared to mock-infected ones (Figure 3B). Moreover, DDC did not colocalize with either DENV NS3 or HCV NS5A, despite the fact that DDC displayed a staining pattern resembling the one of the ER-marker calnexin, in consistence to our previous report [21], and a significant portion of DDC was detected in the membrane cellular fraction.

Interestingly, DENV negatively affects the levels of PI3K-DDC complexes (Figure 4), that we previously showed to be formed by the 50 kDa DDC monomer and the ~60 kDa DDC species interacting with PI3K [21], in co-immunoprecipitation experiments. Thus, PI3K-DDC may be a functional complex playing a significant role in the life cycle of the virus. Moreover, the evidence for the DDC-PI3K interaction in mock-infected hepatocytes coincides well with the localization of the 50 kDa DDC in the membrane fraction of the cell (Figure 3A), as PI3K is known to be recruited in the membranes [104,105]. It is interesting that although the virus downregulates DDC possibly through accumulation of the 50 kDa monomer in the membranes, it does not allow this accumulation to result in increased levels of DDC-PI3K complex.

In turn, the activity of the PI3K/AKT pathway, that is known to contribute to the establishment of DENV acute/apoptotic and HCV chronic/persistent infection [59,62,63,64,65,66], was shown to be important for the virus-mediated effect on the 50 kDa DDC monomer and DDC mRNA (Figure 7 and Figure S8). Interestingly, PI3K inhibition had the same impact on the regulation of DDC exerted by both viruses (Figure 7), although it had opposite results on DENV and HCV viral replication levels [59,65,66,67]. In addition, PI3K inhibition impairs DDC mRNA and total DDC protein expression (Figure 7), in agreement to our previous study [21], through an AKT-independent manner (Figure S8) [106]. This downregulation of *DDC* gene expression upon PI3K inhibition could be attributed to a cell response for survival, since DDC has been implicated in the apoptotic machinery [20]. Thus, we conclude that although PI3K activity could induce DDC expression, viral infection, which at least at the early stages upregulates PI3K, does not allow a PI3K-mediated increase of DDC, in order to ensure cell survival and, subsequently, maintain viral replication.

In addition, our data support that DDC, in turn, inhibits DENV and HCV propagation, as overexpression of DDC protein or treatment with its substrates l-dopa and 5-HTP reduced viral RNA levels (Figure 5 and Figure S7), while the inhibition of DDC enzymatic activity upon treatment with carbidopa significantly enhanced the RNA replication and the released infectivity of both viruses (Figure 6 and Figure S7). In contrast to HCV, the effect of carbidopa on DENV released infectivity was more pronounced than the one observed at the replication, which may be due to the different life cycles of the two viruses and suggest that, in the case of DENV, carbidopa has an additional effect at the post-replication level. Indeed, carbidopa ameliorated the loss of cell viability of DENV-infected cells observed at late hours p.i (Figure 6D right), implying that DDC may be implicated in DENV-triggered cell death. This is in agreement with previous results attributing a possible role of DDC in apoptosis [20]. By increasing cell survival, DDC inhibition may result in sustaining viral RNA replication and enhancing virus production. In the case of HCV, the effect of carbidopa on viral replication and released infectivity occurs concomitantly with an increase in intracellular ATP levels, and thus, it may also be associated with cell viability. This may be related to the carbidopa-mediated activation of PI3K/AKT pathway (Figure 6E), which is expected to positively affect HCV replication. PI3K/AKT activation may also account for the carbidopa-related rescue of cell viability in DENV-infection (Figure 6D). Moreover, carbidopa did not affect the virus-mediated regulation of the 120 kDa DDC complex and the 50 kDa DDC monomer levels, despite the fact that it induced the expression of DDC gene (Figure 6D left) and enhanced the DDC protein levels (Figure 6C,E), in both infected and mock-infected cells. This DDC upregulation, which is consistent with our previous study, occurs also with other DDC inhibitors [102] and can be possibly attributed to feedback mechanisms that induce *DDC* gene transcription to compensate for the loss of DDC function. The effect of carbidopa on DDC expression is opposite than the one exerted by the PI3K inhibitor LY294002, suggesting a negative functional correlation between DDC and PI3K, which was characterized in our previous report [21].

Interestingly, the negative effect of DENV infection on the amount of the PI3K-DDC complex (Figure 4) is similar to the previously reported one exerted by carbidopa [21]. This suggest that the effect of carbidopa on PI3K-DDC complex is probably beneficial for the virus. Specifically, upon carbidopa treatment, the liberation of PI3K from PI3K-DDC complex could permit the activation of PI3K, implying that DDC inhibits PI3K by sequestering it. This is supported by our results showing that carbidopa enhances AKT-phosphorylation and DDC gene expression (Figure 6C,E), which both are PI3K activity-dependent (Figure 7). Thus, carbidopa, through PI3K liberation and activation, could positively affect HCV and DENV replication. In turn, these viruses may aim at the disruption of PI3K-DDC complex in order to increase the activity of the PI3K/AKT pathway.

It is important that analyses of liver biopsy specimens derived from HCV patients with liver fibrosis due to chronic infection, showed a significant inverse correlation between DDC mRNA and HCV genome levels (Figure 8). Moreover, for the specific liver samples, a negative association was observed between the levels of DDC mRNA and of PI3K/AKT downstream targets HK2 and LDH (data not shown). HK2 and LDH were previously shown to positively correlate with HCV RNA [68]. These results are in perfect agreement with data obtained in cell culture, indicating a strong link among viral replication, DDC expression, and PI3K/AKT pathway activity.

In conclusion, although l-dopa decarboxylase has been previously classified as a neglected and misunderstood enzyme, and its physiological function in peripheral tissues still remains unknown [16,17,18], our data provide new evidence about its role as a regulator of DENV and HCV replication in human hepatocytes as well as of DENV-induced cell death. Most importantly, our in vivo data from HCV patients confirmed the inverse correlation between DDC mRNA and HCV RNA levels, which underlines the significance of this biological phenomenon. Thus, our results unravel the importance of DDC in the intricate interaction of DENV and HCV with the host cell and open new possibilities in defining novel therapeutic targets. Moreover, taking into account the aforementioned implication of PI3K/AKT pathway in HCV-DDC relationship and DDC expression, as well as the established role of this pathway in HCC development [107], we hypothesize that DDC might be an important determinant in HCV-induced carcinogenesis.

Finally, although HCV and DENV exhibit significant differences concerning the course of the disease -persistent chronic liver disease and acute/self-limiting infection, respectively, and the effect on cell viability, these differences have no significant role in the interaction of the viruses with DDC. Thus, we suggest that this bidirectional inverse relationship between DDC and viral propagation is probably conserved among the members of the *Flaviviridae* family.

## Supplementary Materials

The following are available online at https://www.mdpi.com/2073-4409/8/8/837/s1, Figure S1. Effect of different DENV titers on DDC regulation, Figure S2. Downregulation of non-neuronal and neuronal DDC transcripts upon DENV-infection, Figure S3. Effect of the HCV RNA replication on DDC protein, Figure S4. Hydrophobicity of DDC protein in DENV- and mock-infected Huh7 cells, Figure S5. Analysis of DENV NS3 protein localization in relation to the ER, Figure S6. Virus replication kinetics, Figure S7. Carbidopa affects at least the viral RNA replication step and DDC substrates inhibit viral replication, Figure S8. Effect of AKT inhibition on DENV- and HCV-mediated DDC protein regulation in infected cells.
