# Supplementary material for: Emerging Role of l-Dopa Decarboxylase in Flaviviridae Virus Infections
Source: Cells. 2019 Aug 5;8(8):837. doi: 10.3390/cells8080837 (PMC6721762; doi:10.3390/cells8080837)
Supplement: Supplementary file 1 [file cells-08-00837-s001.pdf]

Supplementary Data for

Emerging role of L-Dopa decarboxylase in Flaviviridae virus infections

Efseveia Frakolaki [1], Katerina I. Kalliampakou [1], Panagiota Kaimou [1], Maria Moraiti [1], Nikolaos Kolaitis [1], Haralabia Boleti [2], John Koskinas [3], Dido Vassilacopoulou [4] and Niki Vassilaki [1*]

[1] Laboratory of Molecular Virology, Hellenic Pasteur Institute (HPI), 11521, Athens, Greece
[2] Light Microscopy Unit, Hellenic Pasteur Institute, 11521, Athens, Greece
[3] 2nd Department of Internal Medicine, Medical School of Athens, Hippokration Hospital, 11521, Athens, Greece
[4] Section of Biochemistry and Molecular Biology, Faculty of Biology, National and Kapodistrian University of Athens, 15701, Athens, Greece
* Correspondence: nikiv@pasteur.gr; Tel.: +30-210-647-8875

1. Supplementary Materials and Methods

1.1. Antibodies and Chemicals

AKT inhibitor VIII (AKTi-1/2) was obtained from Cayman Chemical and TritonX-114 from Sigma. Staining of the Endoplasmic Reticulum (ER) marker Calnexin was performed by using a rabbit polyclonal antibody (Abcam) at a dilution of 1:500.

1.2. Temperature-Induced Phase Separation in Triton X-114.

The non-ionic detergent Triton X-114 was precondensed prior to use in order to remove possible hydrophilic contaminants. Phase partition was performed according to the method of Bordier [1], as described previously [2]. Briefly, Huh7 cells were trypsinized and resuspended in ice cold TBS (10 mM Tris-HCl, 150 mM NaCl, pH 7.2). Triton X-114 was added to a final concentration of 2%. The mixture was incubated for 15 min on ice with occasional mixing and then centrifuged in a microcentrifuge for 10 min at 4C and 11,752 · g. The pellet (Triton X-114 insoluble/phospholipid rich phase), the upper phase (detergent-depleted) and the lower phase (detergent-enriched) were assayed for Western Blot analysis of DDC.

1.3. DDC mRNA detection cells by RT-PCR

Total RNA isolation from Huh7 cells was performed using TRIzol reagent (Ambion), according to manufacturer's instructions. Reverse transcription using random hexamers (Qiagen) and PCR using primers specific for DDC (Supplementary Table 1) was performed as described previously [3].

1.4. mRNA quantification by RT-qPCR

Total RNA isolation and reverse transcription were performed as described above. Real-time quantitative PCR was performed using KAPA SYBR FAST qPCR Master Mix (Kapa Biosystems) as well as primer pairs specific for the mRNAs of non-neuronal DDC (primers LS1 and T2R, Table 1) and neuronal DDC (primers B and T2R, Table 1) that we have previously described [4,5]. The 14-3-3-zeta polypeptide (YWHAZ) housekeeping gene was used as a normalization control (YWHAZ-F: 5′-GCTGGTGATGACAAGAAAGG-3′ and YWHAZ-R: 5′-GGATGTGTTGGTTGCATTTCCT-3′).

2. Supplementary Data

Supplementary Table 1: Priming oligonucleotides used for RT-PCR analysis of DDC mRNA in Huh7 cells.

Primer Name	Sequence	DDC exon	Primer 5′ position*
LS1 (F)	CTGGCACATGTGGCTGGAAATGC	1 (non neuronal)	-201
B (F)	AACTGTCACTGTGGAGAGGA	1 (neuronal)	-86
DDC1 (F)	AGAGGGAAGGAGATGGTGGATTA	2	25
T2R (R)	GGGGCTGTGCCTGTGCGT	3	222
MZ1R (R)	CACTCCTCCCCCTTCTCC	4	428

* Nucleotide numbers refer to the sequences NM_001082971.1 (non neuronal) and NM_000790.3 (neuronal), in relation to the AUG initiation codon.

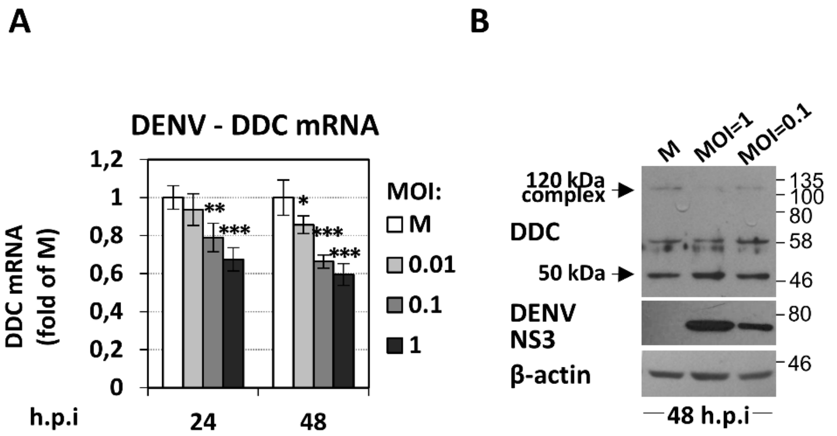

DENV/M	MOI = 1	MOI = 0.1
DDC 120 kDa	0.21 ± 0.01	0.30 ± 0.05
DDC 50 kDa	2.07 ± 0.17	1.48 ± 0.20

Supplementary Figure S1. Effect of different DENV titers on DDC regulation. Huh7 cells were inoculated with cell-culture produced DENV virions at different MOIs (0.01, 0.1 or 1) for 4 h, washed twice with fresh culture medium and further cultured for the indicated hours post-infection (p.i). **(A)** RT-qPCR analysis of total DDC mRNA levels. Values are expressed relative to the ones derived from mock-infected (M) cells at each time-point. YWHAZ mRNA levels were used for normalization. Bars represent mean values from three independent experiments in triplicate. Error bars indicate standard deviations. * $p<0.05$, ** $p<0.01$ and *** $p<0.001$ vs control. **(B)** (Top) Western blot analysis using anti-DDC-CT, anti-PI3K, anti-DENV NS3 or anti-β-actin antibodies. Numbers on the right refer to the positions of molecular mass marker proteins. β-actin was used as loading control. A representative experiment of 3 independent repetitions is shown. (Bottom) Image quantification of DDC signals (mean values from 3 independent repetitions), normalized to β-actin and to the values obtained with mock-infected cells.

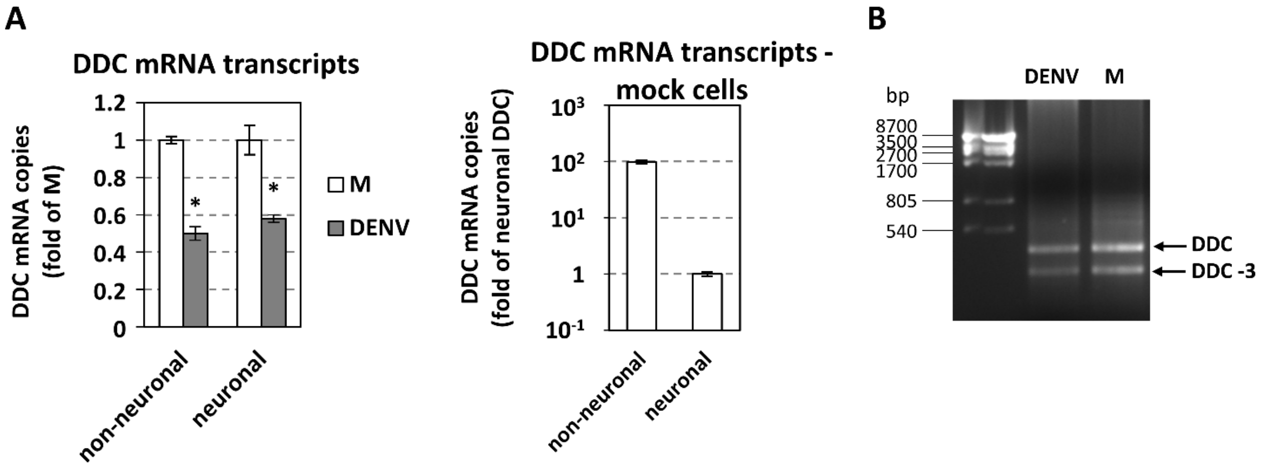

Supplementary Figure S2. (A) Downregulation of non-neuronal and neuronal DDC transcripts upon DENV-infection. RT-qPCR analysis of non-neuronal DDC (primers LS1 and T2R) and neuronal DDC (primers B and T2R) mRNA levels in Huh7 cells infected with DENV (MOI = 1) for 48 h or mock-infected (M) cells. Left: Values from infected cells are expressed for each transcript type relative to the ones derived from mock cells. Right: Relative expression of non-neuronal versus neuronal DDC mRNA. YWHAZ mRNA levels were used for normalization. Bars represent mean values from two independent experiments in triplicate. Error bars indicate standard deviations. **(B) Downregulation of DDC mRNA variants that differ due to alternative splicing of exon 3 upon DENV-infection.** RT-PCR amplification of DDC mRNA in Huh7 cells infected with DENV (MOI = 1) for 48 h and mock-infected cells. Exons 2-4 were amplified using forward primer DDC1 and reverse primer MZ1R and then analyzed by gel electrophoresis.

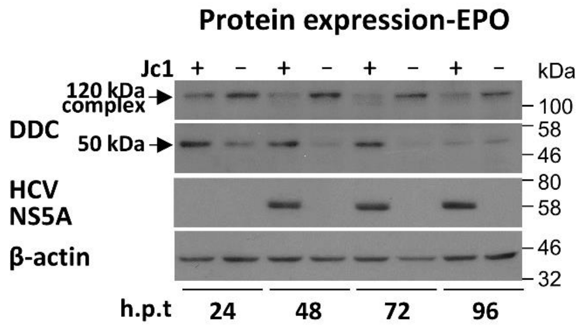

HCV/M	DDC 120 kDa	DDC 50 kDa
**24 h p.i**	0.52 ± 0.07	2.58 ± 0.48
**48 h p.i**	0.34 ± 0.03	2.65 ± 0.27
**72 h p.i**	0.26 ± 0.09	3.04 ± 0.52
**96 h p.i**	0.41 ± 0.08	1.98 ± 0.16

Supplementary Figure S3. Effect of the HCV RNA replication on DDC protein. Huh7-Lunet cells were electroporated with *in vitro* transcribed Jc1 full-length RNA (10 μg RNA/4x10^6 cells) or with a capped-polyadenylated Renilla luciferase expressing RNA (mock-electroporated, M) and cultured for indicated h p.t. (Left) Western blot analysis was performed using anti-DDC-CT, anti-HCV NS5A or anti-β-actin antibodies. β-actin was used as loading control. A representative experiment of 3 independent repetitions is shown. (Right) Image quantification of DDC signals (mean values from 3 independent repetitions), normalized to β-actin and to the values obtained with mock-transfected cells.

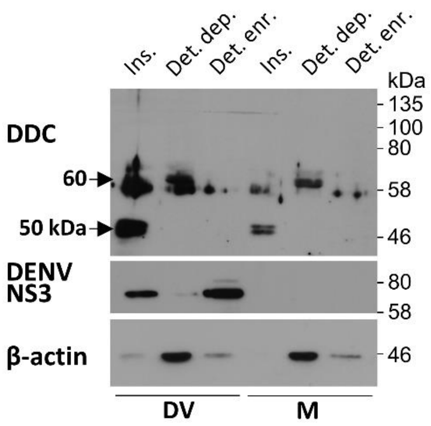

Supplementary Figure S4. Hydrophobicity of DDC protein in DENV- and mock-infected Huh7 cells. Western blot analysis of DDC after SDS-PAGE of lysates of DENV-infected (MOI = 1) for 48 h and mock-infected Huh7 cells subjected in temperature-induced separation using Triton X-114. The distribution of DDC protein species in the phospholipid-rich/Triton X-114 insoluble phase (Ins.), detergent depleted phase (Det. dep.) and detergent enriched phase (Det. enr.) was analyzed using the anti-DDC-CT antibody. The distribution of DENV NS3 was also analyzed. β-actin was used as loading control of infected and mock-infected cells. A representative experiment of 3 independent repetitions is shown.

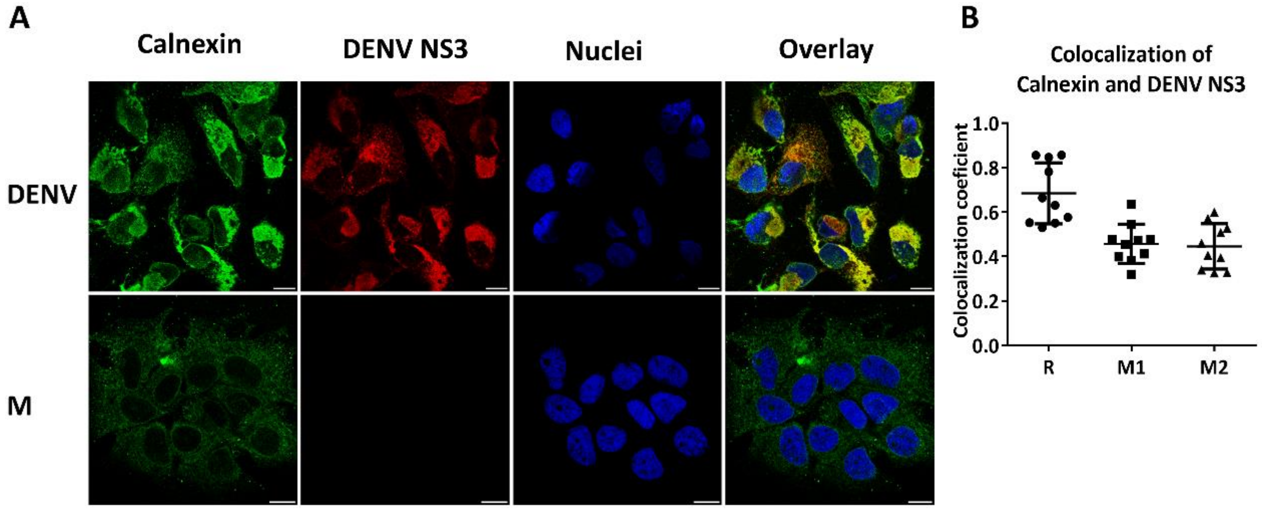

Supplementary Figure S5. Analysis of DENV NS3 protein localization in relation to the ER. (A) Immunofluorescence and confocal microscopy analysis of DENV NS3 protein (red) and ER marker calnexin (green) in Huh7.5 cells infected with DENV (MOI = 1) for 48 h in comparison to mock-infected (M) cells. Nuclei were stained with TO-PRO-3 iodide (blue). On the right, merged images of the green, red and blue fluorescence are shown. Bar, 10 μM. (**B)** Graphical representation of the Pearson correlation coefficient (R) and Manders' colocalization coefficient (M1, M2) values between DENV NS3 and calnexin. Each spot represents a single analyzed infected cell.

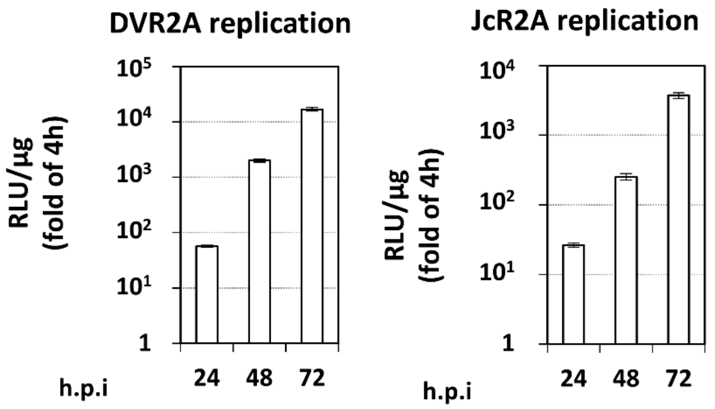

Supplementary Figure S6. Virus replication kinetics in Huh7 cells infected with DVR2A (MOI = 0.1) and in Huh7.5 cells infected with JcR2A (MOI = 0.5). Cells were inoculated with cell culture produced virus for 4 h and lysed at indicated h p.i. Viral replication derived *Renilla* luciferase (R-Luc) activity was measured and expressed as relative light units (RLU) per µg of total protein amount. Values are expressed relative to the ones obtained at 4 h. Bars represent mean values from three independent experiments in triplicate. Error bars indicate standard deviations.

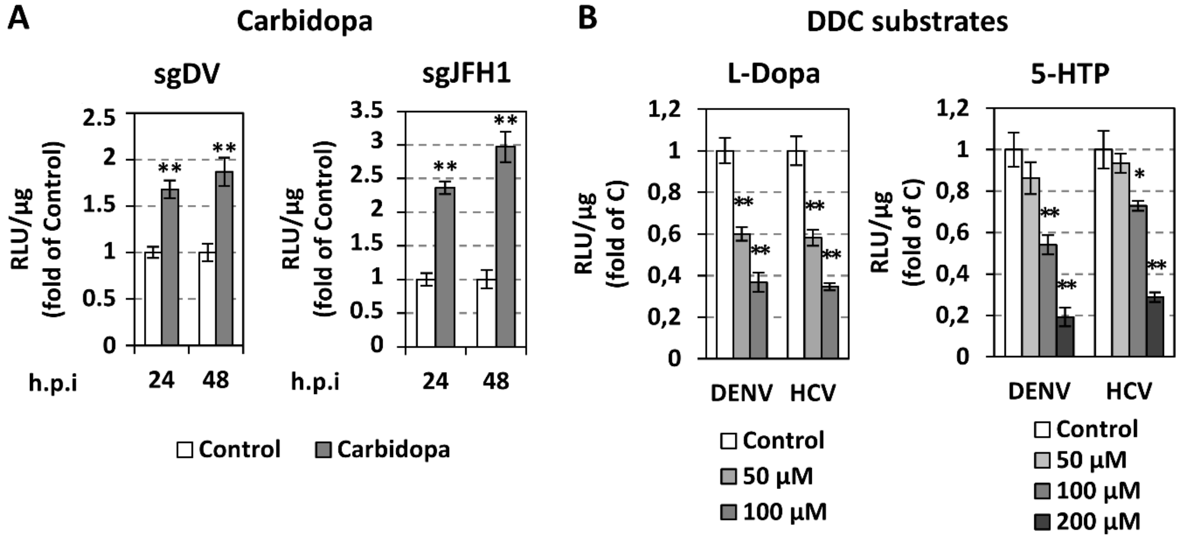

Supplementary Figure S7. A. Carbidopa affects at least the viral RNA replication step. Huh7 and Huh7-Lunet cells were electroporated with *in vitro* transcribed subgenomic reporter DV-2 16681 RNA (left) or HCV JFH1 RNA (right), respectively (10 µg RNA/4x10[6] cells), and treated, or not (control), with 20 µM carbidopa or with the solvent DMSO (control) for the indicated h p.t. Cells were then lysed and virus replication-derived R-Luc activity was determined and expressed as RLU/µg of total protein amount. Values from control cells treated were set to one for each time point. Bars represent mean values from at least three independent experiments in triplicate. ** $p<0.001$ vs control. **B. DDC substrates inhibit viral replication.** Huh7 cells were inoculated with DVR2A and Huh7.5 cells with JcR2A, at MOI = 0.1 for 4 h and then treated or not with the indicated concentrations of L-Dopa or 5-HTP for 48 h or 72 h, respectively for the two viruses. Cells were then lysed and virus replication-derived R-Luc activity was determined and expressed as RLU/µg of total protein amount. Values from control cells treated were set to one for each time point. Bars represent mean values from at least three independent experiments in triplicate. * $p<0.01$ vs control, ** $p<0.001$ vs control.

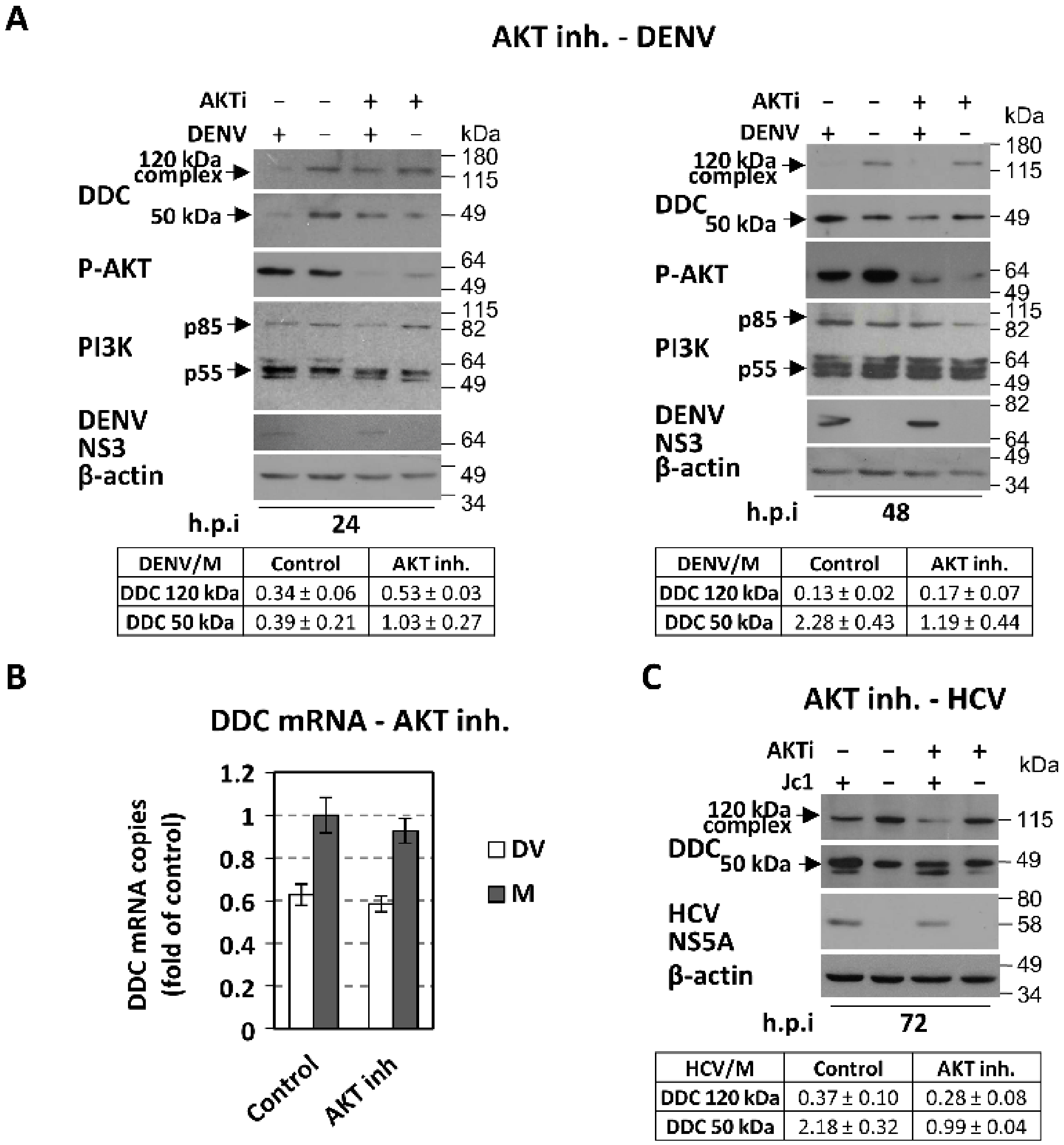

DENV/M	Control	AKT inh.
DDC 120 kDa	0.34 ± 0.06	0.53 ± 0.03
DDC 50 kDa	0.39 ± 0.21	1.03 ± 0.27

DENV/M	Control	AKT inh.
DDC 120 kDa	0.13 ± 0.02	0.17 ± 0.07
DDC 50 kDa	2.28 ± 0.43	1.19 ± 0.44

HCV/M	Control	AKT inh.
DDC 120 kDa	0.37 ± 0.10	0.28 ± 0.08
DDC 50 kDa	2.18 ± 0.32	0.99 ± 0.04

Supplementary Figure S8. Effect of AKT inhibition on DENV- and HCV-mediated DDC protein regulation in infected cells. (A, C) (Top) Huh7 or Huh7.5 cells were mock-infected (M) or inoculated with DENV or Jc1, respectively, at MOI = 1 for 4 h, then treated with the AKT inhibitor VIII (5 μM) for the indicated h p.i. Western blot analysis of cell lysates was performed using anti-DDC-CT, anti-p-AKT, anti-PI3K, anti-DENV NS3 **(A)** or anti-HCV NS5A **(C)** and anti-β-actin antibodies. β-actin was used as loading control. Representative experiments of 3 independent repetitions are shown. (Bottom) Image quantification of DDC signals (mean values from 3 independent repetitions), normalized to β-actin and to the values obtained with mock-infected cells. **(B)** RT-qPCR analysis of DDC mRNA levels was performed in the above Huh7 cells inoculated with DENV at MOI = 1 for 4 h, then treated with the AKT inhibitor VIII (5 μM) and further cultured for 48 h. Values obtained from the control-non treated and mock-infected cells were set to 1. Bars represent mean values from two independent experiments performed in triplicate. Error bars indicate standard deviations.

References

1. Bordier, C. Phase separation of integral membrane proteins in Triton X-114 solution. *J Biol Chem* **1981**, *256*, 1604-1607.
2. Vassiliou, A.-G.; Vassilacopoulou, D.; Fragoulis, E.G. Purification of an Endogenous Inhibitor of L-Dopa Decarboxylase Activity from Human Serum. *Neurochemical research* **2005**, *30*, 641-649, doi:10.1007/s11064-005-2752-7.
3. Vassiliou, A.G.; Siaterli, M.Z.; Frakolaki, E.; Gkogkosi, P.; Paspaltsis, I.; Sklaviadis, T.; Vassilacopoulou, D.; Vassilaki, N. L-Dopa decarboxylase interaction with the major signaling regulator PI3K in tissues and cells of neural and peripheral origin. *Biochimie* **2019**, *160*, 76-87, doi:10.1016/j.biochi.2019.02.009.
4. Kokkinou, I.; Fragoulis, E.G.; Vassilacopoulou, D. The U937 macrophage cell line expresses enzymatically active l-Dopa decarboxylase. *Journal of Neuroimmunology* **2009**, *216*, 51-58, doi:10.1016/j.jneuroim.2009.09.001.
5. Siaterli, M.Z.; Vassilacopoulou, D.; Fragoulis, E.G. Cloning and expression of human placental L-Dopa decarboxylase. *Neurochemical research* **2003**, *28*, 797-803.
